# Explainable artificial intelligence through graph theory by generalized social network analysis-based classifier

**DOI:** 10.1038/s41598-022-19419-7

**Published:** 2022-09-08

**Authors:** Serkan Ucer, Tansel Ozyer, Reda Alhajj

**Affiliations:** 1 The Scientific and Technological Research Council of Turkey, TUBITAK, Ankara, Turkey; 2 Department of Computer Engineering, Ankara Medipol University, Ankara, Turkey; 3 Department of Computer Science, University of Calgary, Alberta, Canada; 4 Department of Computer Engineering, Istanbul Medipol University, Istanbul, Turkey; 5 Department of Heath Informatics, University of Southern Denmark, Odense, Denmark

**Keywords:** Mathematics and computing, Computational biology and bioinformatics

## Abstract

We propose a new type of supervised visual machine learning classifier, GSNAc, based on graph theory and social network analysis techniques. In a previous study, we employed social network analysis techniques and introduced a novel classification model (called Social Network Analysis-based Classifier—SNAc) which efficiently works with time-series numerical datasets. In this study, we have extended SNAc to work with any type of tabular data by showing its classification efficiency on a broader collection of datasets that may contain numerical and categorical features. This version of GSNAc simply works by transforming traditional tabular data into a network where samples of the tabular dataset are represented as nodes and similarities between the samples are reflected as edges connecting the corresponding nodes. The raw network graph is further simplified and enriched by its edge space to extract a visualizable ‘graph classifier model—GCM’. The concept of the GSNAc classification model relies on the study of node similarities over network graphs. In the prediction step, the GSNAc model maps test nodes into GCM, and evaluates their average similarity to classes by employing vectorial and topological metrics. The novel side of this research lies in transforming multidimensional data into a 2D visualizable domain. This is realized by converting a conventional dataset into a network of ‘samples’ and predicting classes after a careful and detailed network analysis. We exhibit the classification performance of GSNAc as an effective classifier by comparing it with several well-established machine learning classifiers using some popular benchmark datasets. GSNAc has demonstrated superior or comparable performance compared to other classifiers. Additionally, it introduces a visually comprehensible process for the benefit of end-users. As a result, the spin-off contribution of GSNAc lies in the interpretability of the prediction task since the process is human-comprehensible; and it is highly visual.

## Introduction

Machine learning (ML) has dominated our daily life with many real-world applications, such as healthcare decision support systems, search engine recommendation systems, and autonomous driving, among others. One of the most common tasks of ML is ‘classification’ which broadly involves two steps, training and testing. While training inspires a model from one part of the data, testing uses the remaining data to check the accuracy of the model which reflects its capability in correctly realizing the classes of new samples. The data types processed with ML are not limited to tabular form, but may exist in a wide range of forms, including multimedia. Still, tabular data may be considered the most common form of data types^1^, dominating most of the applications of ML. The main struggle of the current ML paradigm is explainability of existing ML model to naive users. While the performance of the classification accuracy increases as new models emerge, surprisingly, classification decisions become complex to be justified by humans^2^. This ‘explainability’ phenomenon limits the usage of ML models in critical real-world applications (e.g., law or traffic management) since the context of a decision is hard to be justified and explained to the end-users. Our proposed social network analysis-based visual classifier, GSNAc, aims to solve this problematic aspect of ML by introducing visual cues, both at the model and at the prediction steps.

Social networks form a specific type of network graphs used for depicting real-life paradigms in the form of ‘actors’ and their ‘interactions’ with each other in a closed ecosystem^3^. In its most primitive form, a social network consists of a finite number of nodes (called actors) connected to each other via weighted edges (a weight of one for all edges is also possible) which represent a sort of ‘social interaction’ based on some kind of social quality, e.g., ‘frequency of their coffee meetings’. A pervasive form of a social network is ‘the friendship network’, e.g., Facebook.

Social Network Analysis (SNA) is a study conducted to understand various aspects of a network in general as well as its nodes individually and in groups. For example, in a friendship network, it is possible to find the most influential node (i.e., a person) in the analyzed domain. SNA techniques are widely used in various applications from link prediction for recommending useful connections in a ‘friendship network’ to detecting fraud in a ‘financial transactions network’, among others. SNA techniques mainly include, but are not limited to, detecting node or actor centrality, community (structure) detection, edge capacity, flow, and representative selection.

### An overview of our past works

For a social network, it is not always necessary for the interaction to be linked to a human activity. Indeed, various types of entities other than humans may also socialize, or the relationship between them may be interpreted as a social activity analogous to human activities. Accordingly, interactions can be defined for a broader spectrum of entities such as among genomes, mice varieties, animals, insects, fruit types, etc. In this respect, increasing interest in the SNA domain has allowed many diverse real-life phenomena to be studied. In our former works^3,4^, we tried to address two machine learning tasks using graph theory fundamentals: “feature reduction via SNA” and “the SNAc—classification via SNA of time-genomic datasets”, respectively. For our work described in^5^, we employed SNA techniques and studied specific data from the life sciences domain, which in general do not represent any real ‘social’ value. The techniques themselves proved to be useful in the classification of a tabular dataset. We represented genomic datasets as social network graphs, where genomes are represented as ‘actors’ (nodes) and ‘their vectorial similarity’ to other genomes together with their interaction levels form ‘edges’.

### Current model and the key contributions

Following the success of the proof-of-concept of this approach, we now continue searching for an extended generalized framework that is again based on graph theory and SNA. The target involves addressing the classification of tabular datasets containing either or both numerical and categorical features. Thus, the proposed GSNAc Model is a new type of visually explainable machine learning classifier that works on tabular datasets.

**Figure 1:**
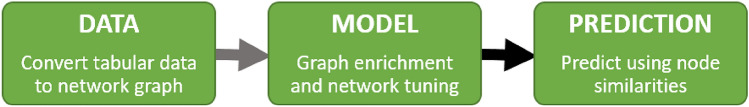
The brief pipeline of the GSNAc model.

Our approach (see Fig. 1) moderately increases the explainability of the whole classification process, since it presents a traceable visual output for each predicted sample. Besides, the ‘graph classifier model—GCM’, which sits at the heart of the classification process, is again in a social network form that makes it visualizable. Those aspects merely address the current problem posed by the most advanced current set of state-of-the-art classifiers, which is the explainability of the classification task (i.e., XAI—explainability of artificial intelligence applications). To summarize, the key aspects and contributions of the GSNAc model could be enumerated as follows:

i. Clarity of the method, explainable process, deterministic classifier,
ii. Graphical interface, explainability, visual engagement,
iii. Versatility (i.e., works well for the classification of both numerical and categorical features),
iv. Superior or on par performance (in terms of prediction accuracy) with the well-known state-of-the-art tabular data classifiers.

### Structure of the research presented

We will present our current work by introducing the problem and the techniques that will be used to address the problem posed in “Related work and key concepts” section. We will then discuss details of the GSNAc Model starting with an overview in “GSNAc: a visual supervised learner” section. We will further detail the model in “The data: converting tabular data to raw network graph”, “The model: graph enrichment and extraction of the graph classifier model”, “The prediction process: using visual graph classifier model (GCM)” sections. Experiments and results of the comparison with conventional machine learning classifiers will be present in “Experiments and results” section. We dedicated “Explainability of the GSNAc model” section to elaborate on the explainability aspect of the proposed GSNAc Model. Discussion and further study are covered in “Discussion and further work” section.

In Appendix I, we provide the code, the detailed parameters of the classifiers & the datasets, and the runtime parameters of the GSNAc Model, including the raw performance results, and the URL to the directory of outputs GSNAc produced throughout the experiments, all for the sake of the reproducibility of the conducted research. Finally, in Appendix II, we provide the user interface and the output of GSNAc.

## Related work and key concepts

### Graphs, social networks, and SNA

Social networks^6^ form a subtype of network graphs that originated from graph theory. Graph theory is briefly the study of network graphs, which are mathematical representations used to model pairwise relations between objects. A graph is normally characterized by a set of nodes and edges which connect nodes. Typically, edges may have a default weight of 1; alternatively, varying weights may be assigned to various edges to reflect the value of the relationship between the connected nodes. Indeed, many real-world problems and systems can be reduced and represented as a network graph. For instance, the traffic system connecting roads in an area may be reorganized as a graph where intersections and roundabouts act as nodes and edges represent roads connecting them. This way, a navigation map application may identify a possible shortest path between two locations by analyzing the graph to determine the most appropriate route based on the combination of the costs of road segments connecting some consecutive junctions.

A network graph is simply the combination of nodes and edges connecting nodes. Each edge has a source node, a target node, and a weight value showing its effect based on the criteria applied to construct the network. Assume an edge e1 between nodes a and b has weight 4, and another edge e2 between a and c has weight 3, then we conclude that a is more similar to b than c. In some cases, edges of a network graph can be directed, i.e., the direction of connection between the source node and the target node matters, e.g., in a directed graph, two edges e1 from a to b and e3 from b to a are two different edges. If the direction doesn’t matter in the domain of study, it is safer to use undirected networks.

Having originated from the social sciences domain, Social Network Analysis (SNA) is the sum of measures inspired from graph theory to analyze the relationship among social entities. Social Network analysis encompasses many measures that can be broadly grouped as:

- Centrality studies for nodes
- Edge analysis
- Node distance-similarity methods
- Community structure studies

SNA measures used in this study are node similarity analysis, edge analysis and centrality studies.

### Machine learning: the classification task

It is very common to come across the results of scientific experiments involving measurements of various aspects (in other words, features) of objects in a specific domain, and at the end objects are often categorized into a limited number of cases (called classes).

In the machine learning domain, the term ‘classification’ implies the task of first learning the characteristics of a group of objects from specific classes and then predicting the correct class for some previously unseen objects forming the test data.

**Figure 2:**
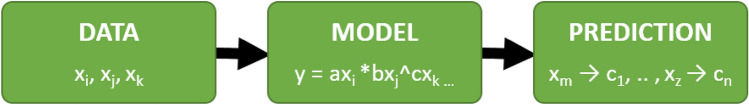
Illustrative box diagram of a general machine learning classification model.

Any learner model follows almost the same black-box model shown in Fig. 2. According to this generalization, it starts with the ‘Data’ box, where the dataset under study is examined in terms of quality, completeness, and complementarity of its feature space and sample data points. Tasks such as decomposition, transformation, elimination, and improvement of the feature space are some of the common methods used in this step.

The aim of the ‘classifier model’ box is to find and describe the connection between the data and its class, by employing mathematical and statistical techniques. Finally, in the ‘Prediction’ box, the proposed model implements its technique to process data with the model presented to produce appropriate predictions. Crucially, in this step ‘benchmarking’ ensures that we assess the merit of the classifier. Here, internal and external benchmarking can be employed, i.e., ‘statistical hypothesis testing techniques’ as the internal evaluation, and comparing the performance of the proposed classifier with the state-of-the-art established models as the external evaluation.

There is no silver bullet technique for classification tasks^7^. For instance, some classification techniques may perform efficiently on biological data while may fail to produce meaningful predictions on financial data. State-of-the-art techniques for tabular data classification consider decision tree-based classifiers. Among those, notably XGBoost^8^ depends on tree learning, and dominates most of the recent^9^ data science competitions. Another recent and popular research is deep learning, where its main contribution is on feature space reorganization and selection. Despite the fact that deep learning could be used for classification, their effective usage is still mainly on text, image, and video data types^10^.

### Machine learning and network graphs

Even though SNA techniques are widely used to solve various problems from engineering to media; machine learning classification using network graphs is a domain that has not received enough attention from the research community. In our former work described in^5^, we employed various SNA techniques to convert time-sequential genomic data into a social network and developed a classification model to predict classes. The method proved to be useful when compared to conventional algorithms, but was limited to being applied only on time-sequential data. Within this work, we aim to generalize our classification model (abbreviated as GSNAc) to an extent that the method can be applicable to numerical/categorical datasets which include class information.

The machine learning applications for the social network domain are generally centered around two topics^11^: (i) the similarity between two graphs (or subgraph matching), and (ii) the similarity of the nodes/edges in a graph. The first one aims to find a motif between possibly two different-sized graphs (in terms of nodes and edges) to deduct a result, e.g., a deduction that both graphs have similar network dynamics. Here, a recent work in this area is worth mentioning^12^. It employed graph theory techniques for text classification. In^12^, NLP researchers first convert sentences into adjacency matrices based on the co-occurrence of words, and then to graphs. The process then efficiently predicts word similarities by employing graph similarity techniques. At this point, we state that the proposed GSNAc model has no relation with this ‘inter-graph similarity’ aspect of the mentioned research domain.

On the other hand, finding possible similarities ‘within’ a graph, in terms of the similarity between nodes or edges, is a problem partially addressed by GSNAc. The ‘node classification’ problem refers to the process of propagating labels to the unlabelled nodes on a partially labeled graph by evaluating the similarity of nodes^13,14^. A recent research in this area (refer to^15^) handles node classification problems by introducing graph neural networks over graphs (particularly Graph Convolutional Networks—GCNs). This approach basically learns hidden layer representations that encode both the local graph structure and features of nodes. It has been proved useful in predicting labels on some types of networks including citation networks where it predicts the type of the document. Within this context, the node classification problem partially matches the definition of the problem tackled by GSNAc; since (after converting data to a graph and after its enrichment) we also predict the class (i.e., label) of an unseen node by evaluation against labels of the training nodes. However, in comparison to the node classification problem setting, there are major challenges that GSNAc needs to deal with. First, in the node classification problem, the complete (weighted) graph (including all nodes—i.e., training and test parts) is already in place initially, making it easier to assign labels based on their topological properties based on the complete connection structure within all nodes (labeled or unlabelled). Second, node classification can predict labels iteratively by depending on known positions, possibly starting with a frequently labeled portion of the graph. We believe that these dynamics differentiate the two problems well enough. Here it is worth mentioning that the GSNAc model is actually an end-to-end machine learning classifier on its own.

To sum up, we would like to present a recent trend on converting tabular data into networks. This approach becomes pretty useful when the aim is to visualize data to get an intuition-based idea. A recent research described in^16^ efficiently converts tabular data into images (rather than networks as GSNAc does), and predicts drug responses of gene expression profiles using deep learning techniques on the generated images. While this approach does not coincide with GSNAc, it shows the popularization of expressing tabular data as 2D visible graphic items, a reasonable step towards explainability of tabular learning.

## GSNAc: a visual supervised learner

In this section, following the formal problem description, we will present an overview of the GSNAc model both in diagram and pseudocode formats. A detailed description of the methodology and parameter selection strategies will be covered in the following three subsections.

### Problem description and naming conventions

The formal problem description of GSNAc is exactly the same as the problem definition of machine learning for the categorical classification task: given a multi-class tabular dataset with numerical and categorical features, the process involves learning from the seen data and predicting the class of unseen data. Multi-class classification refers to predicting one or more classes for each sample. Imbalanced classification refers to a classification task where the distribution of samples across the classes is skewed. In other words, most of the existing objects represent specific classes. Fortunately, GSNAc is not merely a bi-class classifier. It does not aims to work only on balanced data, it also works on imbalanced data which makes it useful on a broader spectrum of problems. A final note on the use of GSNAc is that it currently does not support regression prediction which is predicting continuous numerical values.

**Figure 3:**
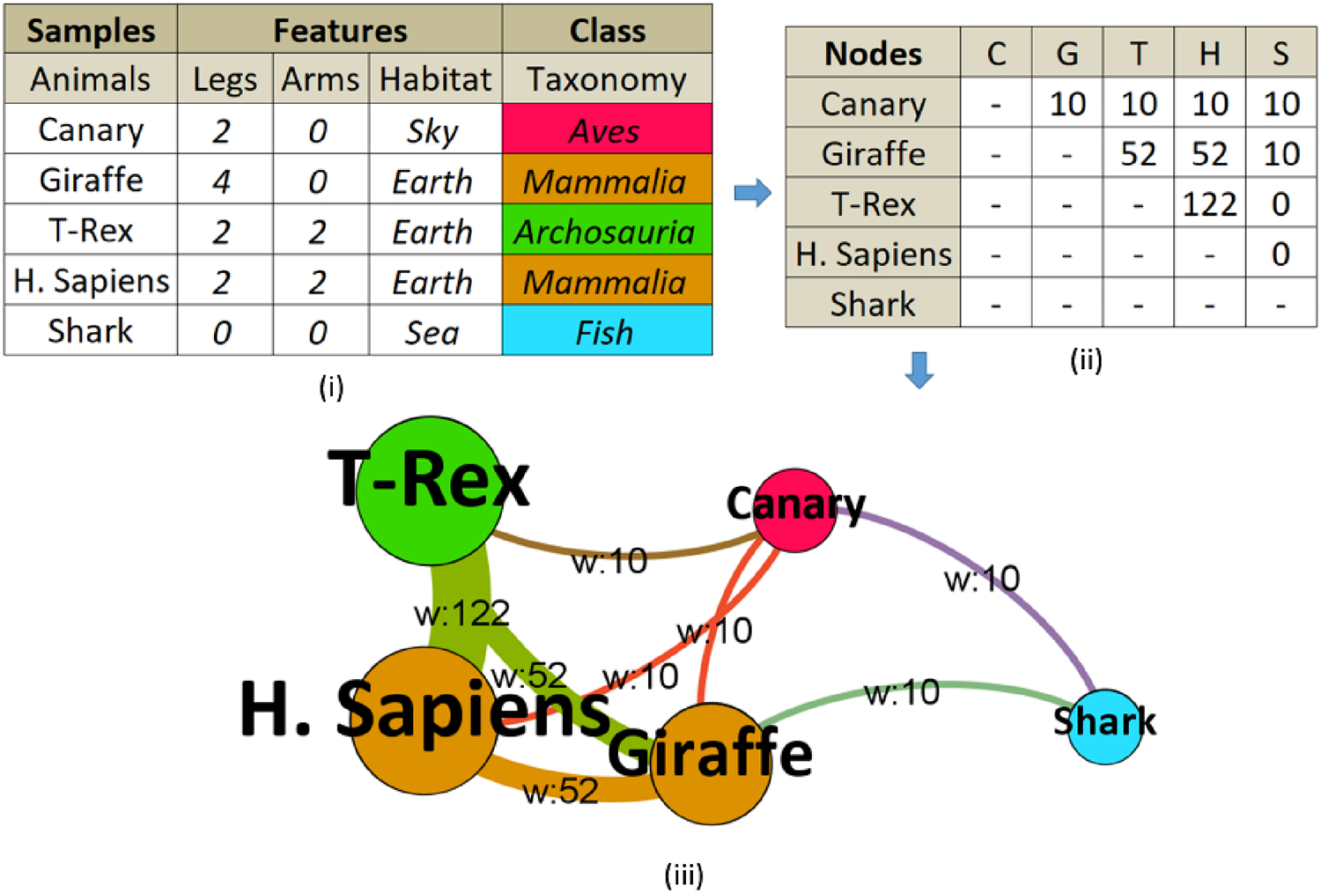
(i) Tabular data converted to adjacency (similarity) matrix (ii), and graph (iii) constructed and enriched with node degrees, edge weights, and colored by communities (classes). Layout graph intuitively shows the importance of some nodes (i.e., H. Sapiens and T-Rex have the highest number of weighted connections, therefore, larger in size) and visually depicts how similar nodes are (e.g., Shark has the smallest similarity to other nodes so it is distant from the core).

Before we delve into detailed descriptions of the GSNAc methodology, we would like to present (see Fig. 3) the ‘naming’ convention for this research. Throughout this work, for the dataset part, our preference is to call individual observations (of an experiment) as the ‘samples’, and their defining characteristics (i.e., predictors) are called ‘features’ of the samples. Categories of the results of the experiment are called ‘classes’.

After converting the dataset to a network graph, basically dataset ‘samples’ become ‘nodes’ in the graph, and dataset ‘features’ are blended into ‘edges’ of the graph. A node may have a ‘neighbor’ if there is an edge between them. Finally, the classes are represented in the network graph as the ‘communities’.

**Figure 4:**
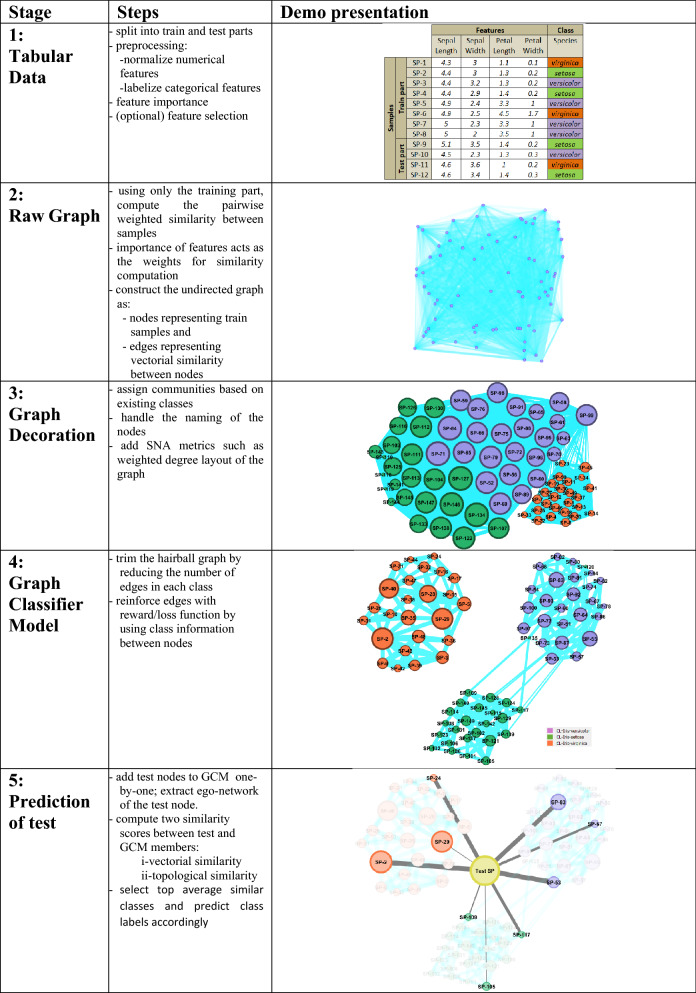
General overview of the GSNAc model illustrated on Iris dataset.

The pipeline of the GSNAc methodology is diagrammatically described in Fig. 4. It starts with the preprocessing of raw tabular data. After data is separated into training and test parts, a raw graph is generated using only the training part of the data. This raw graph is decorated and further processed to achieve a leaner version called graph classifier model (GCM) with fewer edges and repositioned nodes.

This GCM was then employed for predicting the class of test samples using two different similarity scores. Next, we present the master pseudocode summarizing the GSNAc methodology.
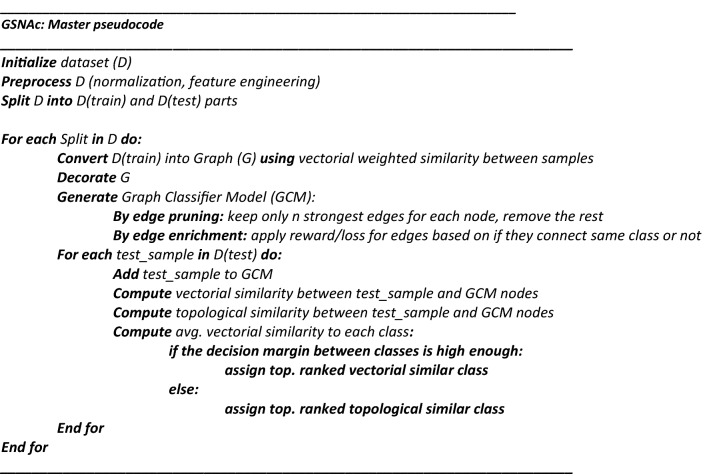


In Fig. 1 we described the GSNAc Models’ process pipeline: this type of framing comes useful when proposing a novel model. In this sense, we organized the following three sections focused on the boxes of the model, namely ‘Data: Graph generation’, ‘Model: GCM Generation’ and ‘Class Prediction’.

## The data: converting tabular data to raw network graph

In this subsection, we present details on how we process the dataset, turn it into a network graph and finally how we produce, and process features that belong to the graph. Topics to be covered are:

- splitting the data,
- preprocessing,
- feature importance and selection,
- computation of similarity between samples, and
- generating of the raw graph.

### Splitting the tabular data

After preprocessing the data, the next step is to split the dataset into training and test samples for validation purposes. We selected cross-validation (CV) as the validation method since it is the de facto standard in ML research. For CV, the full dataset is split into k folds; and the classifier model is trained using data from (k-1) folds then tested on the remaining k’th fold. Eventually, after k iterations,, average performance scores (like F1 measure or ROC) of all folds are used to benchmark the classifier model.

A crucial step of CV is selecting the right proportion between the training and test subsamples, i.e., number of folds. Determining the most appropriate number of folds k for a given dataset is still an open research question^17^, besides de facto standard for selecting k is accumulated around k = 2, k = 5, or k = 10. To address the selection of the right fold size, we have identified two priorities:

- Priority 1—Class Balance: We need to consider every split of the dataset needs to be class-balanced. Since the number of class types has a restrictive effect on selecting enough similar samples, detecting the effective number of folds depends heavily on this parameter. As a result, whenever we deal with a problem which has low represented class(es), we selected k = 2.
- Priority 2—High Representation: In our model, briefly, we build a network from the training subsamples. Efficient network analysis depends on the size (i.e., number of nodes) of the network. Thus, maximize training subsamples with enough representatives from each class (diversity) is our priority as much as we can when splitting the dataset. This way we can have more nodes. In brief, whenever we do not cross priority 1, we selected k = 5.

By balancing these two priorities, we select efficient CV fold size by evaluating the characteristics of each datasets in terms of their sample size and the number of different classes. The selected fold value for each dataset will be specified in the “Experiments and results” section. To fulfill the class balancing priority, we employed stratified sampling. In this model, each CV fold contains approximately the same percentage of samples of each target class as the complete set.

### Feature space organization and preprocessing

Preprocessing starts with the handling of missing data. For this part, we preferred to omit all samples which have one or more missing feature(s). By doing this, we have focused merely on developing the model, skipping trivial concerns.

As stated earlier, GSNAc can work on datasets that may have both numerical and categorical values. To ensure proper processing of those data types, as a first step, we separate numerical and categorical features^18^. First, in order to process them mathematically, categorical (string) features are transformed into unique integers for each unique category by a technique called labelization. It is worth noting that, against the general approach, we do not use the one-hot-encoding technique for transforming categorical features, which is the method of creating dummy binary-valued features. Labelization does not generate extra features, whereas one-hot-encoding extend the number of features.

For the numerical part, as a very important stage of preprocessing, scaling^19^ of the features follows. Scaling is beneficial since the features may have a very different range and this might affect scale-dependent processes like distance computation. We have two generally accepted scaling techniques which are normalization and standardization. Normalization transforms features linearly into a closed range like [0, 1], which does not affect the variation of values among features. On the other hand, standardization transforms the feature space into a distribution of values that are centered around the mean with a unit standard deviation. This way, the mean of the attribute becomes zero and the resultant distribution has a unit standard deviation. Since GSNAc is heavily dependent on vectorial distances, we do not prefer to lose the structure of the variation within features and this way our choice for scaling the features becomes normalization. Here, it is worth mentioning that all the preprocessing is applied on the training part of the data and transformed on the test data, ensuring no data leakage occurs.

### Feature importance and selection

Feature Importance (FI) broadly refers to the scoring of features based on their usefulness in prediction. It is obvious that in any problem some features might be more definitive in terms of their predictive capability of the class. Moreover, a combination of some features may have a higher effect than others in total than the sum of their capacity in this sense. FI models, in general, address this type of concern. Indeed, almost all ML classification algorithms use a FI algorithm under the hood; since this is required for the proper weighting of features before feeding data into the model. It is part of any ML classifier and GSNAc. As a scale-sensitive model, vectorial similarity needs to benefit much from more distinctive features.

For computing feature importance, we preferred to use an off-the-shelf algorithm, which is a supervised ‘k-best feature selection’^18^ method. The K-best feature selection algorithm simply ranks all features by evaluating features’ ANOVA analysis against class labels. ANOVA F-value analyzes the variance between each feature and its respective class and computes F-value which is the ratio of the variation between sample means, over the variation within the samples. This way, it assigns F values as features’ importance. Our general strategy is to keep all features for all the datasets, with an exception for genomic datasets, that contain thousands of features, we practiced omitting. For this reason, instead of selecting some features, we prefer to keep all and use the importance learned at this step as the weight vector in similarity calculation.

### Computation of similarity between samples and construction of the raw graph

In this step, we generate an undirected network graph G, its nodes will be the samples and its edges will be constructed using the distance metrics^20^ between feature values of the samples. Distances will be converted to similarity scores to generate an adjacency matrix from the raw graph. As a crucial note, we state that since we aim to predict test samples by using G, in each batch, we only process the training samples.

In our study for constructing a graph from a dataset we defined edge weights as the inverse of the Euclidean distance between the sample vectors. Simply, Euclidean distance (also known as L2-norm) gives the unitless straight line (shortest) distance between two vectors in space. In formal terms, for f-dimensional vectors *u* and *v*, Euclidean distance is defined as:

$$d\left(u,v\right)=\sqrt[2]{\sum_{f}{\left({u}_{i}-{v}_{i}\right)}^{2}}$$

A slightly modified use of the Euclidean distance is introducing the weights for dimensions. Recall from the discussion of the feature importance in the former sections, some features may carry more information than others. So, we addressed this factor by computing ‘a weighted’ form of L2 norm based on distance which is presented as:

$${dist\_L2}_{w}\left(u,v\right)=\sqrt[2]{\sum_{f}{{w}_{i}({u}_{i}-{v}_{i})}^{2}}$$

where w is the n-dimensional feature importance vector and *i* iterates over numerical dimensions.

The use of the Euclidean distance is not proper for the categorical variables, i.e. it is ambiguous and not easy to find how much a canary’s habitat ‘sky’ is distant from a sharks’ habitat ‘sea’. Accordingly, whenever the data contains categorical features, we have changed the distance metric accordingly to L0 norm. L0 norm is 0 if categories are the same; it is 1 whenever the categories are different, i.e., between the ‘sky’ and the ‘sea’ L0 norm is 1, which is the maximum value. Following the discussion of weights for features, the L0 norm is also computed in a weighted form as ${dist\_L0}_{w}\left(u,v\right)=\sum_{f}{w}_{j}(({u}_{j}\ne {v}_{j})\to 1)$, where *j* iterates over categorical dimensions.

After computing the weighted pairwise distance between all the training samples, we combine numerical and categorical parts as: ${{dist}_{w}\left(u,v\right)}^{2}={{dist\_L2}_{w}\left(u,v\right)}^{2}+ {{dist\_L0}_{w}\left(u,v\right)}^{2}$. With pairwise distances for each pair of samples, we get a *n* x *n* square and symmetric distance matrix D, where n is the number of training samples. In matrix D, each element shows the distance between corresponding vectors.

$$D= \left[\begin{array}{ccc}0& \cdots & d(1,n)\\ \vdots & \ddots & \vdots \\ d(n,1)& \cdots & 0\end{array}\right]$$

We aim to get a weighted network, where edge weights represent the ‘closeness’ of its connected nodes. We need to first convert distance scores to similarity scores. We simply convert distances to similarities by subtracting the maximum distance on distances’ series from each element.

$$similarity\_s(u,v)=\mathrm{max}\_\mathrm{value}\_\mathrm{of}(D)-{dist}_{w}\left(u,v\right)$$

Finally, after removing self-loops (i.e. setting diagonal elements of A to zero), we use adjacency matrix A to generate an undirected network graph G. In this step, we delete the lower triangular part (which is symmetric to the upper triangular part) to avoid redundancy. Note that, in transition from the adjacency matrix to a graph, the existence of a (positive) similarity score between two samples u and v creates an edge between them, and of course, the similarity score will serve as the ‘vectorial weight’ of this particular edge in graph G.

$$A= \left[\begin{array}{ccc}-& \cdots & s(1,n)\\ \vdots & \ddots & \vdots \\ -& \cdots & -\end{array}\right]$$

The raw graph generated in this step is a ‘complete graph’: that is, all nodes are connected to all other nodes via an edge having some weight. Complete graphs are very complex and sometimes impossible to analyze. For instance, it is impossible to produce some SNA metrics such as betweenness centrality in this kind of a graph.

## The model: graph enrichment and extraction of the graph classifier model

In this section, we first compute basic SNA metrics over the network graph, then decorate the raw graph with certain properties of the tabular data, and finally process edge analysis of the raw graph. To reduce the complexity and denseness of the graph, we will trim unimportant edges by considering their structure to get a subtle form of the graph which will serve as our visual classifier model.

**Figure 5:**
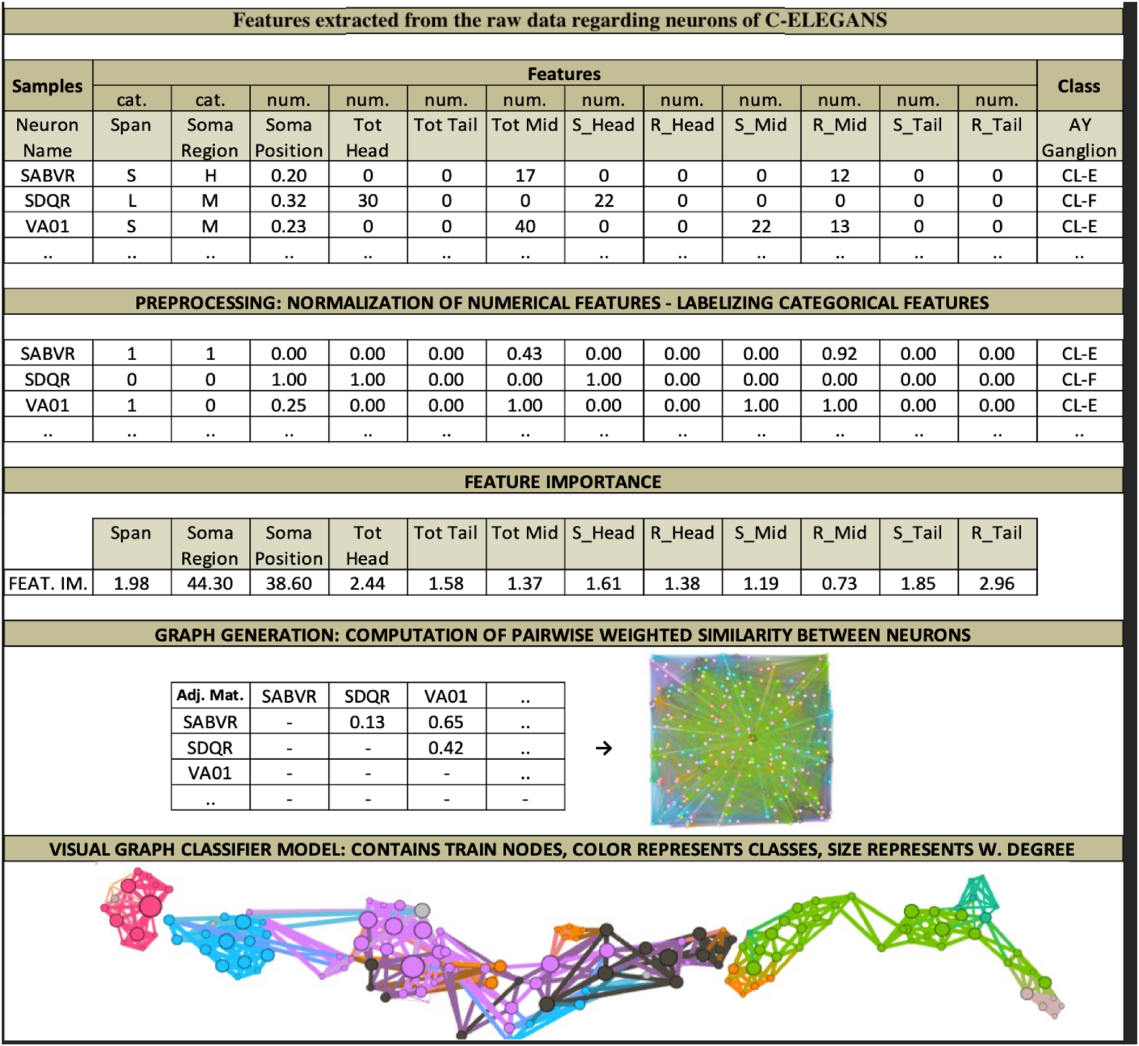
From raw data to GCM demonstrated on *C. elegans* Dataset.

To increase the understandability of the procedures presented, we visualized the whole process in Fig. 5, which belongs to a biological dataset. This dataset inspired from C. Elegans Connectome (https://commons.wikimedia.org/wiki/File:Caenorhabditis_elegans_hermaphrodite_adult-en.svg) incorporates 279 samples over 12 features (2 categorical, 10 numerical). It has 10 (imbalanced) classes. C. Elegans is indeed a simple worm, and its whole neuron system has been extracted, filed, and widely researched in recent years^21–23^. This worm has exactly 302 neurons (having unique names and positions) connected to 158 muscles and organs on its body. Out of those 302 neurons, following the Connectome research^24^, non-pharynx neurons and neurons with no synaptic connections are omitted, leaving 279 somatic neurons out of the initial 302. This dataset is useful in showing the capabilities of the proposed model. We choose the ‘AY Ganglion’ feature as the class to be predicted. In this specific case, we intend to find a predictive relationship between the neurons and their connections to specific regions throughout the worm’s body.

### Graph decoration and preprocessing

#### Node naming

In general, the dataset associated with classification has dedicated sample names, e.g., names of the specific neurons in Connectome dataset or CustomerID’s in Caravan Insurance dataset^25^. Usually, this potentially beneficial information is ignored during the classification. In contrast to this, for the sake of explainability, we save and assign sample names throughout our raw graph as node names.

#### Community partition assignment

Community detection in a network graph refers to finding partitions of the graphs (subgraphs of connected nodes) displaying similar characteristics. It is beneficial since it reveals hidden relations among nodes in a network. Inevitably, we need to incorporate community detection within the raw graph. While many algorithms have been developed to detect communities, e.g.,^26–28^, we do skip employing them intentionally since our network is already partitioned based on the given set of classes. That is, since the raw graph is only composed of training samples, we already do know the classes they have and the communities they belong to. In this fashion, we assign classes as communities in the raw graph.

#### Centrality SNA metrics

Within a graph, finding how central a node is (compared to other nodes topologically) in terms of the capacity of its entropy is an important branch of SNA metrics as mentioned in “Related work and key concepts” section. Among other centrality metrics^3^, the most primitive is ‘degree centrality’ which refers to the number of edges connected to a given node. ‘Weighted degree’ is the improved form of degree centrality, which also considers edge weights when aggregating node connections. Despite not using centrality metrics in the GSNAc model in a predictive capacity, we still find them useful in the model to enrich the displays produced. For this purpose, we use ‘weighted degree centrality’ for scaling the size of the nodes. The latter is a visually engaging concept and gives comparative clues about the entropy capacity of a node within a graph.

### Edge pruning

We already highlighted the need for removing self-loops in the former section to simplify the complexity of the raw graph. But, still, this graph is a complete graph, i.e., every node is connected to every other node leading to a graph density of 100%. Now we need to identify and further remove unimportant edges of this complete graph to ensure that our model includes only relevant and important edges; this way, its entropy increases.

Some research^29,30^ already addressed the raffination of the so-called ‘hairball graph’ which is overly dominated by unimportant edges. We prefer to use a custom raffination at this step, keeping only at most k strongest edges for each node and dropping the remaining edges. This way, by keeping the k value small, we can achieve a raffinated, less dense version of the raw graph. Also, by only keeping the most information-bearing edges, entropy of the graph will increase.

Instead of setting a constant k value for each dataset, we have set an upper limit, and dynamically identified the value of k by analyzing the frequency of the least represented class within the population.

### Edge fortification

So far, we have only described how the ‘class’ information of tabular data is only used in graph community assignments. GSNAc might incorporate class information in the model generation step, since it is a supervised model. We prefer to use this very important class information as a secondary step in a reward/loss scheme for edges. This time, we reconsider weights of the edges by analyzing classes of their endpoints. The weight of an edge is improved by a constant factor when it connects two nodes from the same class. Likewise, the same factor is used to decrease the weight of an edge when its endpoints are from different classes. After these steps, the graph may become disconnected since some nodes can lose all of their edges and become isolated, or some group of nodes can be only connected to themselves, creating an isolated connected component.

## The prediction process: using visual graph classifier model (GCM)

As described in the previous sections, we have carefully constructed a vectorial similarity-based network graph, pruned its edges by their importance, and finally generated a leaner graph which will act as our classifier model named GCM. At this point, we will feed test nodes one-by-one into GCM and conduct the prediction by analyzing most similar nodes. For this similarity analysis, we have employed two complementary methods: vectorial and topological.

### Adding test node to GCM

#### Vectorial similarities of test node to GCM nodes

Vectorial similarity is the weighted (L2 and/or L0) distance-based similarity score already computed during the network generation phase. To calculate this vectorial similarity score for each test data to be predicted, upon normalization of test nodes vector, L2 norm-based distance for the numerical part, and L0 norm-based distance for categorical part of the test data are calculated using the same weights that were learned from the training data at step FI. These distance scores are calculated pairwise between the test node and all nodes of GCM. Following the conversion from distances to similarity scores, the test node becomes connected to all nodes of GCM via different edge weights.

#### Topological similarities of test node to GCM nodes

As we already have a topological structure in the form of a graph, we can also analyze the neighborhood structure of nodes. A topological similarity, in its simple form, can be calculated by comparing the neighborhood structure of two nodes: if they have the same neighborhood with similar weights, they can be considered topologically similar. Pairwise cosine similarity^31^ has been used to compute the topological similarity between the test node and nodes of GCM; this measure is useful when one needs to compare two bags of words (with frequencies). Indeed, it is widely used in natural language processing for comparing text similarity.

$$\mathrm{cos}\_similarity(test,v)= \frac{top\_k\_neighbours\_of\_test *neighbours\_of\_v}{\Vert top\_k\_neighbours\_of\_test\Vert *\Vert neighbours\_of\_v\Vert })$$

Our usage of cosine similarity is to define a bag of words as neighborhood lists of nodes and frequencies as the weights of edges (i.e., vectorial similarity) connecting them. As can be seen in the formula, it is again a pairwise calculation as the vector product of the test node (test) and a node from GCM (v) divided by the product of L2 norms of the same vectors. Note that, the value k in the formula refers to the number of neighbors of v; it is expected to be smaller than the number of neighbors of the test node which is already connected to all nodes of GCM.

To conclude, in topological similarity, we do not only consider the direct neighbors of a node from GCM. We also compare two nodes’ neighborhood structure, i.e., how their indirect neighbors match.

### Prediction strategy: assigning class to the test node

Topological similarity is somewhat dependent on vectorial similarity since the former uses edge weights (i.e., vectorial similarity) as input. However, topological similarity does not produce the same graph as vectorial similarity, and GCM built by topological similarity gives some clue about nodes’ position within the graph. In this respect, it can be used as a secondary similarity metric, complementing vectorial similarity. We have decided to blend these two similarity metrics to achieve a higher prediction performance and it turned out that using topological similarities as secondary ‘sage’ (whenever decision margin is small with vectorial similarity) has been proven beneficial for prediction as described next.

**Figure 6:**
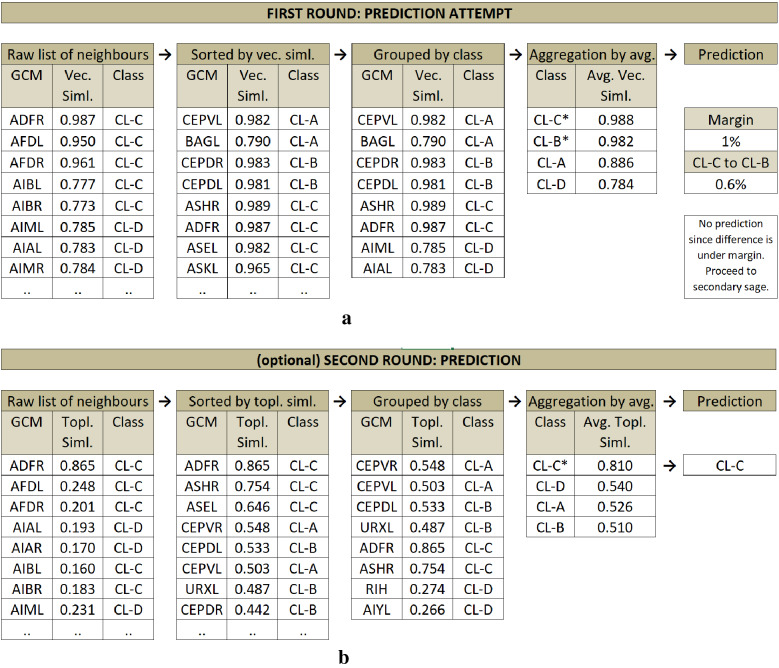
(**a**) Prediction Phase 1 sample diagram on C. Elegans dataset. İllustrating the prediction attempt of the test node ADFL (not displayed in the diagram). Raw list of neighbors of ADFL listed, sorted by vectorial similarity, grouped by class, and finally similarities are aggregated by class. A prediction is not made since the top two highest averaged classes have very close scores (under margin). (**b**) Prediction Phase 2 sample diagram on C. Elegans dataset. Illustrating the prediction of the test node ADFL (not displayed in the diagram). This time, the prediction is made based on topological similarity in the same fashion with the first round. This time, without seeking a distinctive margin, the top (highest topological similarity average) class is assigned as the prediction.

For actual prediction, we primarily use vectorial similarity and keep the maximum number of edges for a given test node from each represented class in GCM based on their weights. Our prediction is then to group and aggregate those edges class-wise to get an average similarity of the test node to each class, respectively (see Fig. 6a). To assign a class to a test node, we only consider the top two high-ranked classes and compare their aggregated similarity scores.

When the top most classes differ by a magnitude, we assign the top-ranked class to the test node. However, in case the vectorial similarity-based model fails to find two distinctively separated classes (i.e., setting a margin threshold of 1% means the difference of the scores of the top two classes must be higher than 1%) we leave the prediction completely to the secondary sage, i.e., the topological similarity (see Fig. 6b). This time, in the same fashion, we aggregate the average similarities of the classes over topological similarities, and (without further check) we assign the top-ranked class to the test node.

## Experiments and results

### The datasets

GSNAc was initially designed to work efficiently with balanced and bi-class datasets (i.e., binary classification). However, during the development, we discovered that it can effectively classifies some multiclass and imbalanced datasets, as well. As a proof of concept for GSNAc, we carefully selected as many real-world diverse datasets from various domains with various feature and class structures. We also considered including synthetic datasets as they give the chance to compare similar work by other researchers. Finally, we considered some other custom datasets compiled for this research. Table 1 summarizes the datasets used in the experiments.

**Table 1:** The datasets used in the experiments.

Dataset name	Domain	Samples		Features			Classes	
		Sample Size	CV for splitting	Numerical	Categorical	Total	Class labels	Class distribution
Caravan insurance	Finance	983	5	81	4	85	2	Imbalanced
Iris	Biology	149	5	4	0	4	3	Balanced
Connectome	Biology	279	2	10	2	12	10	Imbalanced
Colon	Medicine	62	2	1988	0	1988	2	Imbalanced
Lymphoma	Medicine	96	2	4026	0	4026	9	Imbalanced
PBC	Medicine	276	2	16	2	18	4	Imbalanced
Heart Kaggle	Medicine	299	5	10	2	12	2	Imbalanced
Heart UCI	Medicine	302	5	6	7	13	2	Balanced
COVID	Medicine	436	2	36	1	37	2	Imbalanced
Breast cancer wisconsin	Medicine	569	5	30	0	30	2	Imbalanced
Pima diabetes	Medicine	768	5	8	0	8	2	Imbalanced
Voice	Signal processing	474	5	20	0	20	2	Balanced
Forest type	Signal processing	497	5	10	44	54	7	Imbalanced
Digits	Signal processing	1797	5	64	0	64	10	Balanced
Wine	Statistics	178	5	13	0	13	3	Balanced
Titanic	Statistics	183	5	4	3	7	2	Balanced
weather rain	Statistics	226	5	16	5	21	2	Imbalanced
Pokerhand	Statistics	250	2	0	10	10	6	Imbalanced
Make blobs	Syntethic	300	5	2	0	2	2	Balanced
Make moons	Syntethic	500	5	2	0	2	2	Balanced

In the preprocessing steps, we standardized the datasets before feeding them to the ML classifiers since some classifiers (especially those based on scale sensitive distance metrics like SVM and kNN) heavily depend on the data to be scaled. To select the standardization over other competing techniques, normalization is also needed especially for SVM RBF classifier (GSNAc’s main competitors are reported in Table 3) since they assume that all the features are centered around zero and variance of the data is of the same order^32^.

### Experimental setup

We implemented the GSNAc Model using Python. We selected the platform of GSNAc to be compatible with python’s sklearn library^33^ since it is the de facto standard platform in the ML domain. Also, it allows parameter optimization by grid or random search methods, making it useful to find the best performer set of parameters for a given task. Within this context, we did not practice parameter search or optimization for this research in order to present a fair comparison with other ML classifiers. We also used other python libraries such as networkx, bokeh, pandas, and NumPy.

### Classifiers used for benchmarking

To be comprehensive and fair in the testing, we decided to include as diverse ML models as possible in terms of their approach for the classification. All the classifiers run with their default parameter set (except for ANN where the maximum number of iterations was upgraded from 200 to 2000 since it does not usually converge with 200 iterations), and likewise, we used default parameters for GSNAc, i.e., we did not search for the best parameters. We used scikit-learn^33^ implementation of the utilized classifiers except xgboost, where we used the native xgboost python library^8^. We present in Table 2 the list of classifiers and their parameters used for the comparison.

**Table 2:** The classifiers used in the experiments.

Classifier name	Classifier type	Parameters
AdaBoost	Ensemble-tree based	base_estimator = None, n_estimators = 50, learning_rate = 1.0, algorithm = 'SAMME.R', random_state = None
Artificial neural networks (ANN)	Function based	max_iter = 2000, hidden_layer_sizes = (100,), activation = 'relu', *, solver = 'adam', alpha = 0.0001, batch_size = 'auto', learning_rate = 'constant', learning_rate_init = 0.001, power_t = 0.5, shuffle = True, random_state = None, tol = 0.0001, verbose = False, warm_start = False, momentum = 0.9, nesterovs_momentum = True, early_stopping = False, validation_fraction = 0.1, beta_1 = 0.9, beta_2 = 0.999, epsilon = 1e−08, n_iter_no_change = 10, max_fun = 15,000
Decision tree	Hierarchical	criterion = 'gini', splitter = 'best', max_depth = None, min_samples_split = 2, min_samples_leaf = 1, min_weight_fraction_leaf = 0.0, max_features = None, random_state = None, max_leaf_nodes = None, min_impurity_decrease = 0.0, class_weight = None, ccp_alpha = 0.0
Naive Bayes Gaussian	Statistical	priors = None, var_smoothing = 1e−09
Gaussian process RBF kernel	Statistical	kernel = RBF, optimizer = 'fmin_l_bfgs_b', n_restarts_optimizer = 0, max_iter_predict = 100, warm_start = False, copy_X_train = True, random_state = None, multi_class = 'one_vs_rest', n_jobs = None
k nearest neighbours (kNN)	Statistical	n_neighbors = 5, *, weights = 'uniform', algorithm = 'auto', leaf_size = 30, p = 2, metric = 'minkowski', metric_params = None, n_jobs = − 1
Random forest	Ensemble-tree based	n_estimators = 100, *, criterion = 'gini', max_depth = None, min_samples_split = 2, min_samples_leaf = 1, min_weight_fraction_leaf = 0.0, max_features = 'auto', max_leaf_nodes = None, min_impurity_decrease = 0.0, bootstrap = True, oob_score = False, n_jobs = None, random_state = None, verbose = 0, warm_start = False, class_weight = None, ccp_alpha = 0.0, max_samples = None
SVM Linear Kernel	Function based	C = 1.0, kernel = 'linear', degree = 3, gamma = 'scale', coef0 = 0.0, shrinking = True, probability = False, tol = 0.001, cache_size = 200, class_weight = None, verbose = False, max_iter =—1, decision_function_shape = 'ovr', break_ties = False, random_state = None
SVM RBF kernel	Function based	C = 1.0, kernel = 'rbf', degree = 3, gamma = 'scale', coef0 = 0.0, shrinking = True, probability = False, tol = 0.001, cache_size = 200, class_weight = None, verbose = False, max_iter =—1, decision_function_shape = 'ovr', break_ties = False, random_state = None
XGBoost	Ensemble-tree based	default parameter set

### Performance comparison of GSNAc with traditional tabular learners

In order to test classification efficiency of the GSNAc on the given datasets, we have investigated the comparison of SNAc with traditional machine learning classification algorithms. We selected ten traditional machine learning classification methods to compare their performance with the GSNAc Model over the twenty datasets listed in Table 1.

Machine learning classification algorithms usually fall into 3 categories, namely statistical, function-based and tree-based (hierarchical) models. We selected at least one algorithm from each category in order to present a balanced comparison.

The reported results, as summarized in Table 3, show that GSNAc beats or on par with other classifiers on 10 of the datasets. Most notable success of these is on the PBC dataset^34^, where the problem is to predict one of 4 heavily imbalanced classes. On the remaining 10 other datasets, where GSNAc doesn’t score at top, we have noted comparable performance with other classifiers.

**Table 3:** Performance comparison of GSNAc with other classifiers. F1 stands for F1 weighted score.

Dataset	Decision Tree		Gaussian RBF		NB Gaussian		AdaBoost		kNN		Random Forest		XGBboost		ANN-MLP		SVM Linear		SVM RBF		GSNAc	
	F1	Rank	F1	Rank	F1	Rank	F1	Rank	F1	Rank	F1	Rank	F1	Rank	F1	Rank	F1	Rank	F1	Rank	F1	Rank
Colon	0.7913	6	0.2828	11	0.7979	5	0.7163	8	0.6696	9	0.8193	4	0.7892	7	0.8234	3	**0.8540**	**1**	0.6262	10	**0.8540**	**1**
Connectome	0.7887	3	0.5919	7	0.5133	10	0.3932	11	0.5391	8	0.7795	4	0.8032	2	0.7430	5	0.6118	6	0.5191	9	**0.8050**	**1**
COVID	0.7074	10	0.7420	7	0.7296	9	0.7394	8	0.6506	11	0.7558	4	0.7660	2	0.7514	5	0.7642	3	0.7509	6	**0.7700**	**1**
Forest type	0.5713	2	0.5657	6	0.4116	11	0.4319	10	0.5458	9	0.5675	5	0.5545	8	0.5596	7	0.5692	3	0.5677	4	**0.6192**	**1**
Iris	0.9329	9	0.9530	3	0.9530	4	0.9259	11	0.9530	2	0.9396	8	0.9262	10	0.9465	7	0.9530	5	0.9466	6	**0.9597**	**1**
Make blobs	0.7100	11	0.7867	3	0.7733	5	0.7499	9	0.7533	8	0.7599	7	0.7266	10	0.7833	4	0.7667	6	0.7867	2	**0.7900**	**1**
Make moons	0.9920	8	0.9740	9	0.8800	11	0.9960	7	**1.0000**	**1**	0.9980	6	**1.0000**	**1**	**1.0000**	**1**	0.8800	10	**1.0000**	**1**	**1.0000**	**1**
PBC	0.3949	10	0.4530	5	0.2544	11	0.4290	8	0.4772	3	0.4332	7	0.4241	9	0.4522	6	0.4898	2	0.4707	4	**0.4947**	**1**
Pokerhands	0.3729	10	0.4415	2	0.3890	9	0.4020	7	0.4157	6	0.4188	4	0.3609	11	0.3942	8	0.4288	3	0.4169	5	**0.4438**	**1**
Weather rain	0.7364	10	0.7419	9	0.5808	11	0.8076	4	0.7454	8	0.8463	2	0.8279	3	0.7916	6	0.7476	7	0.8030	5	**0.8472**	**1**
Titanic	0.7388	7	0.7339	8	0.6380	11	**0.7705**	**1**	0.7596	3	0.7561	4	0.7426	5	0.7070	10	0.7397	6	0.7315	9	0.7700	2
Heart UCI	0.7618	11	0.7810	9	0.7859	8	0.8040	7	0.8145	4	0.8047	5	0.8044	6	0.7713	10	**0.8271**	**1**	0.8172	3	0.8176	2
Heart kaggle	0.7502	9	0.6910	11	0.7540	8	0.7945	6	0.7131	10	**0.8271**	**1**	0.8189	4	0.7865	7	0.8259	2	0.8039	5	0.8193	3
Caravan insurance	0.8759	10	0.8973	7	0.6098	11	0.9065	5	0.9089	3	0.9063	6	0.9089	2	0.8968	8	0.8940	9	**0.9109**	**1**	0.9089	3
Lymphoma	0.6768	7	0.0074	11	0.4587	10	0.5638	9	0.7269	5	0.7600	4	0.7144	6	**0.9256**	**1**	0.9087	2	0.6631	8	0.9060	3
Voice	0.9578	6	0.9557	9	0.8945	11	0.9557	7	0.9430	10	0.9599	5	0.9641	3	0.9662	2	0.9557	8	**0.9705**	**1**	0.9620	4
Breast cancer wisconsin	0.9193	11	0.9665	6	0.9296	10	0.9665	7	0.9646	8	0.9612	9	0.9700	4	0.9753	3	0.9753	2	**0.9771**	**1**	0.9683	5
Digits	0.8523	9	0.9674	7	0.7840	10	0.2633	11	0.9766	3	0.9760	4	0.9644	8	0.9710	6	0.9788	2	**0.9805**	**1**	0.9755	5
Wine	0.9214	10	0.9717	5	0.9719	4	0.9046	11	0.9605	8	0.9719	3	0.9494	9	0.9832	2	0.9605	7	**0.9832**	**1**	0.9661	6
Pima diabetes	0.6904	11	0.7471	6	0.7514	5	0.7393	8	0.7320	9	0.7571	2	0.7275	10	0.7530	4	**0.7677**	**1**	0.7560	3	0.7436	7

The bold values indicates the top score and rank for the respective dataset. Rows are sorted by GSNAc Models’ success, columns (classifiers) are sorted by their respective cumulative successes.

Detailed performance results of GSNAc in comparison with other classifiers are presented in Appendix I.

## Explainability of the GSNAc model

Considering the rise of deep learners, ML models are increasingly getting complex in terms of their prediction model generation. The so-called black-box classifier models are not comprehendible for end-users on how the actual prediction is decided. For instance, a person may be interested in learning the reason for declining his/her loan application which was evaluated by an AI system.

**Figure 7:**
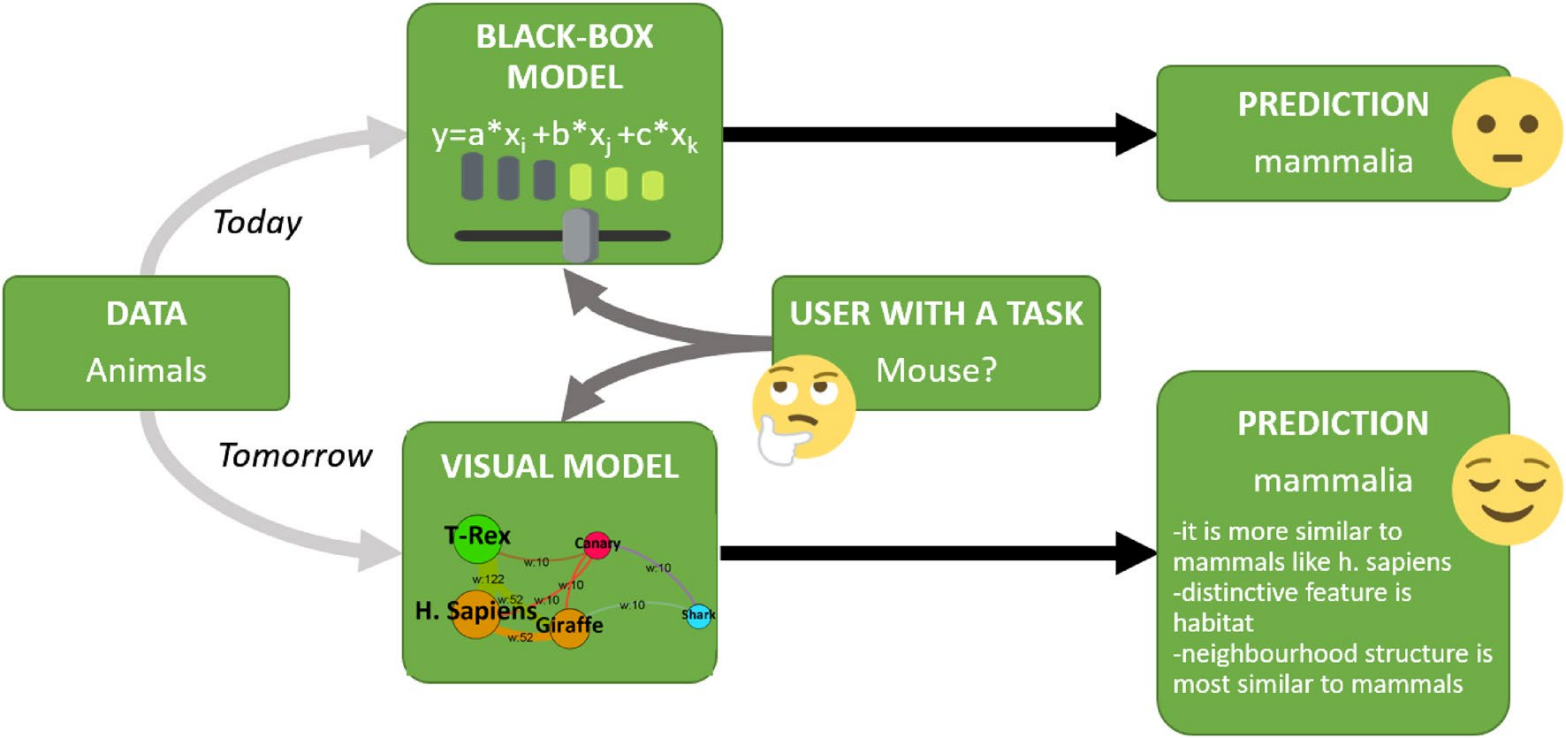
Explainable AI approach versus todays’ classifier models in a nutshell.

Explainable AI (XAI) is a recent trend in machine learning research^2,35^ which aims to identify how predictions are made in an explainable manner (see Fig. 7). Explainability and/ or interpretability is essential for end-users to effectively trust, and manage artificial intelligence applications^36^.

There are various (recent) research efforts conducted to address the explainability of the already existing machine learning classifier models. In^37^, researchers efficiently extended the use of a convolutional neural network classifier by developing an explainability module. With this extra module, they displayed comprehensionable prediction results of microseismic wave-form signal data while keeping excellent classification performance. Similarly, in^38^, researchers put significant effort to translate deep neural networks-based prediction results over traffic analysis data into visually comprehensible map data; thus providing an explainable (learned) trajectory segmentation to the end user of the deep learner model. Though relevant, we argue that these efforts do not actually coincide with the work developed in this research since GSNAc’s procedures and predictions are already designed with explainability in mind, and hence need no further translation procedure for explainability purposes.

**Figure 8:**
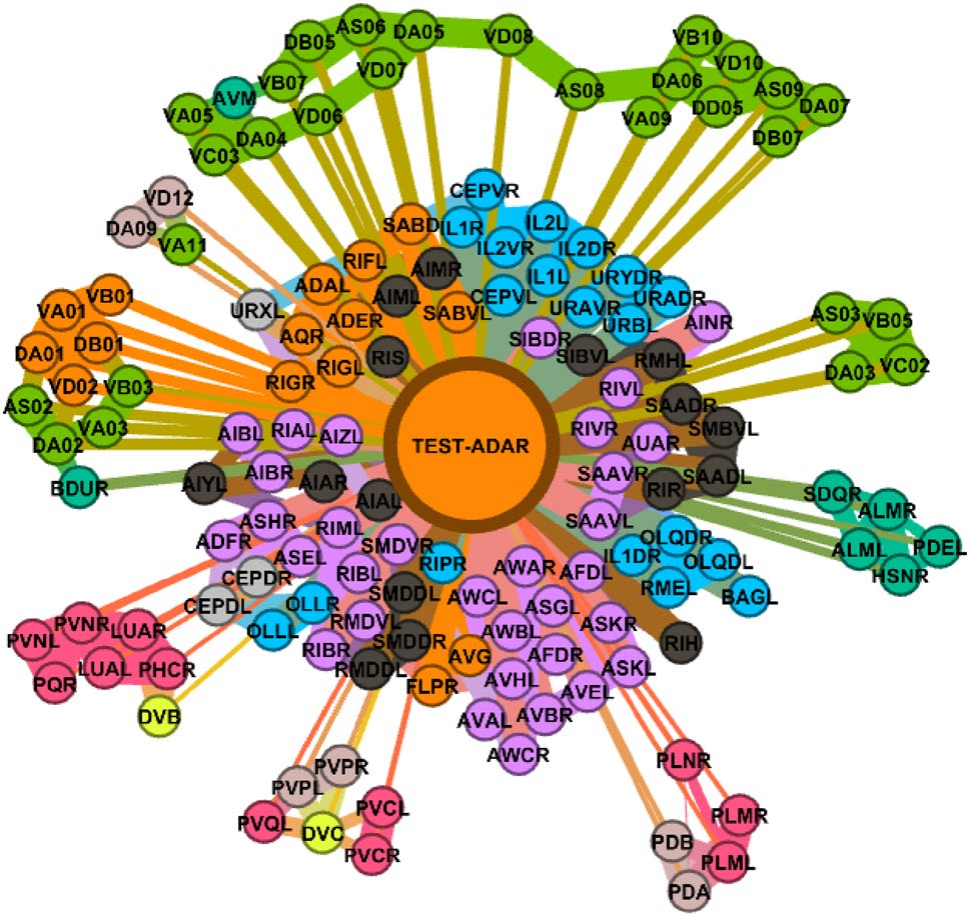
Sample visualization of the prediction step. Test node ADAR from the *C. elegans* dataset has been classified as CL-E (orange).

**Figure 9:**
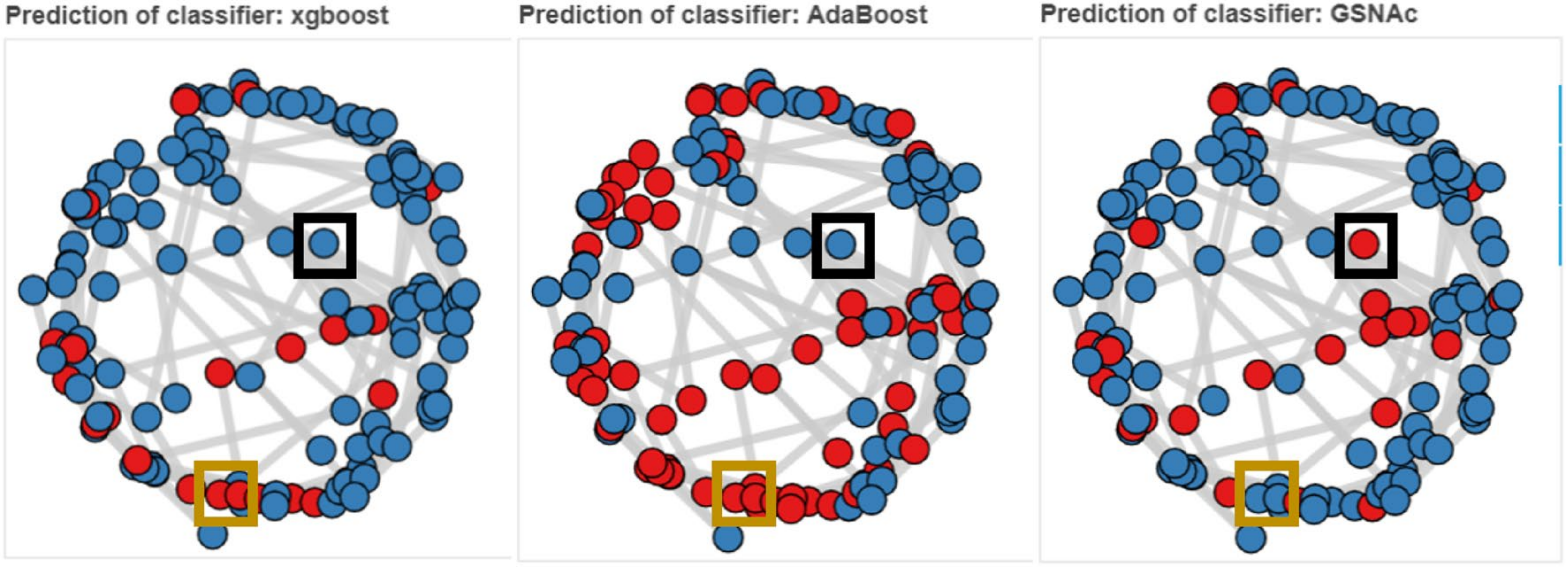
Overall classification result on C. Elegans dataset. Blue color implies that the specific sample has been predicted true by the respective classifier (such as AdaBoost); red color indicates false prediction. Boxes in black indicate cases where the same sample has been predicted true (as in blue colors) by XGBoost and AdaBoost classifiers, but false (as in red colors) by GSNAc, and boxes in orange indicate opposite cases (i.e., false by XGBoost and AdaBoost while true by GSNAc).

SNA concentrates on generating, and visualizing interactions and information flows among network actors. The power of social networks is stemming mostly due to their capacity in revealing and visualizing interactions in a connected structure. It is no doubt that one can comprehend data more efficiently in a visual way, and network graphs allow us to better understand how nodes are similar to each other, which nodes bear most information, and most importantly, how information flows within a network. By converting and visualizing tabular data, we can reveal and learn several aspects, such as: which samples can distribute information to the largest audience, who is connected to the most influential nodes, and who creates fragility to graph, i.e., the single point of failure by being a single connector between two separate communities. As powered up by the idea of network graphs, GSNAc surely presents some advantages of social networks to its users as briefly summarized in the following three stages:

i. The first advantage is the visual classifier model (i.e., GCM) which makes sense of the data by keeping sample names as node names and keeping class information as a colored community. This is illustrated in Figs. 3, 4, and 5.
ii. The second advantage towards explainability lies in the actual visual prediction step (see Fig. 8): end-users can receive visual clues on how the test node is assigned into one class but not others by analyzing the structure of GCM nodes and weights of the edges.
iii. The final advantage is based on the comparison of prediction results of GSNAc across different classifiers (see Fig. 9). GSNAc produces a set of graphs and displays the comparison on the overall prediction process. This way, an end-user can perceive how classifiers (in contrast to GSNAc) predicted a certain sample, whether true or false.

## Discussion and further work

We have proposed GSNAc as a novel graph-based machine learning classifier which works on both numerical and categorical tabular datasets. It outperforms the previous version named SNAc both in performance and capability of handling a wide range of datasets. The main contribution of GSNAc is its capability of visualizing the decision-making process behind the model, while maintaining a fine prediction performance when compared to other classifiers.

GSNAc uses easy-to-grasp similarity-based metrics in graph generation and prediction steps; so might be a choice of some group of end-users who would like to diagrammatically perceive the whole classification process. Since GSNAc also provides prediction margins to end-user, one of its other advantages might be seen as determining and transferring low-margined classification decisions to a human expert for further investigation.

We demonstrated that GSNAc outperforms or is on par with state-of-the-art classifiers across different domains in terms of prediction performance. However, the main drawback of GSNAc is the duration of its run, since its core data structure is a ‘graph’ which has a processing complexity of O(n^2^). Except for scalability, we are not aware of any shortcomings of this Model.

Lastly, we believe in the future this work can be effectively extended into node classification problems.

## Supplementary Information

Supplementary Information.

## Data Availability

The datasets used and/or analysed during the current study available from the corresponding author on reasonable request.

## Abbreviations

XAI: Explainable artificial intelligence
GSNAc: Generalized social network analysis-based classifier
GCM: Graph classifier model
SN: Social network
SNA: Social network analysis
SNAc: Social network analysis-based classifier
ML: Machine learning
NLP: Natural language processing
