# Supplementary material for: Explainable artificial intelligence through graph theory by generalized social network analysis-based classifier
Source: Sci Rep. 2022 Sep 8;12:15210. doi: 10.1038/s41598-022-19419-7 (PMC9458666; doi:10.1038/s41598-022-19419-7)
Supplement: Supplementary file 1 — Supplementary Information. [file 41598_2022_19419_MOESM1_ESM.docx]

# Appendix I

## GSNAc Code, Datasets experimented and detailed output files of the experiments

[*https://drive.google.com/drive/folders/1h1cdiLsGfWZi2G2w4140iUQik_dtTahh?usp=sharing*](https://drive.google.com/drive/folders/1h1cdiLsGfWZi2G2w4140iUQik_dtTahh?usp=sharing)

## A-I- Table 1 - GSNAc Experiments (including parameters used) Overview

| Dataset | CV | Seed Number | Feature Importance Model | Feature Selection Method | Distance to Similarity Method | GCM Kernel Strategy | Maximum Edges in Kernel for Each Class | Maximum Nodes in Kernel for Each Class | Keep Top n Edges to Test |
| --- | --- | --- | --- | --- | --- | --- | --- | --- | --- |
| breast_cancer_wisconsin | 5 | 0 | k_best_based | none | max | keep_n_strongest | 5 | 0 | 5 |
| caravan | 5 | 0 | k_best_based | none | max | keep_n_strongest | 5 | 0 | 5 |
| colon | 2 | 0 | k_best_based | none | max | keep_n_strongest | 5 | 0 | 5 |
| connectome | 2 | 0 | k_best_based | none | max | keep_n_strongest | 5 | 0 | 5 |
| covid | 2 | 0 | k_best_based | none | max | keep_n_strongest | 5 | 0 | 5 |
| digits | 5 | 0 | k_best_based | none | max | keep_n_strongest | 5 | 0 | 25 |
| forest_type | 5 | 0 | k_best_based | none | max | keep_n_strongest | 5 | 0 | 5 |
| heart_kaggle | 5 | 0 | k_best_based | none | max | keep_n_strongest | 5 | 0 | 5 |
| heart_uci | 5 | 0 | k_best_based | none | max | keep_n_strongest | 5 | 0 | 25 |
| iris | 5 | 0 | k_best_based | none | max | keep_n_strongest | 5 | 0 | 25 |
| lymphoma | 2 | 0 | k_best_based | jenkspy | max | keep_n_strongest | 5 | 0 | 5 |
| make_blobs | 5 | 0 | k_best_based | none | max | keep_n_strongest | 5 | 0 | 25 |
| make_moons | 5 | 0 | k_best_based | none | max | keep_n_strongest | 5 | 0 | 25 |
| pbc | 2 | 0 | k_best_based | none | max | keep_n_strongest | 5 | 0 | 5 |
| pima_diabetes | 5 | 0 | k_best_based | none | max | keep_n_strongest | 5 | 0 | 5 |
| pokerhand | 2 | 0 | k_best_based | none | max | keep_n_strongest | 5 | 0 | 5 |
| titanic | 5 | 0 | k_best_based | none | max | keep_n_strongest | 5 | 0 | 25 |
| voice | 5 | 0 | k_best_based | none | max | keep_n_strongest | 5 | 0 | 25 |
| weather_rain | 5 | 0 | k_best_based | none | max | keep_n_strongest | 5 | 0 | 5 |
| wine | 5 | 0 | k_best_based | none | max | keep_n_strongest | 5 | 0 | 25 |

## A-I- Table 2 Full Report of Classification Performance

| **Selected Dataset** | **Ranking** | **Classifier** | **CV Value** | **Seed Number** | **Precision** | **Recall** | **F1-Score (weighted)** | **# Samples (Support)** | **# Features** | **# Classes** |
| --- | --- | --- | --- | --- | --- | --- | --- | --- | --- | --- |
| colon | 1 | SVM Linear | 2 | 0 | 0.85373347 | 0.85483871 | 0.85404685 | 62 | 1988 | 2 |
| colon | 1 | GSNAc | 2 | 0 | 0.85373347 | 0.85483871 | 0.85404685 | 62 | 1988 | 2 |
| colon | 3 | ANN - MLP | 2 | 0 | 0.82472039 | 0.82258065 | 0.82342906 | 62 | 1988 | 2 |
| colon | 4 | Random Forest | 2 | 0 | 0.82027086 | 0.82258065 | 0.81929607 | 62 | 1988 | 2 |
| colon | 5 | Naive Bayes Gaussian | 2 | 0 | 0.80724053 | 0.80645161 | 0.79792317 | 62 | 1988 | 2 |
| colon | 6 | Decision Tree | 2 | 0 | 0.79275003 | 0.79032258 | 0.79132526 | 62 | 1988 | 2 |
| colon | 7 | xgboost | 2 | 0 | 0.78846802 | 0.79032258 | 0.78917878 | 62 | 1988 | 2 |
| colon | 8 | AdaBoost | 2 | 0 | 0.71705672 | 0.72580645 | 0.71629446 | 62 | 1988 | 2 |
| colon | 9 | kNN | 2 | 0 | 0.67919178 | 0.69354839 | 0.66964221 | 62 | 1988 | 2 |
| colon | 10 | SVM RBF | 2 | 0 | 0.66382915 | 0.67741935 | 0.6261725 | 62 | 1988 | 2 |
| colon | 11 | Gaussian RBF | 2 | 0 | 0.77747403 | 0.40322581 | 0.2827744 | 62 | 1988 | 2 |
| connectome | 1 | GSNAc | 2 | 0 | 0.81343821 | 0.8172043 | 0.80502032 | 279 | 13 | 10 |
| connectome | 2 | xgboost | 2 | 0 | 0.80846484 | 0.82437276 | 0.80319948 | 279 | 13 | 10 |
| connectome | 3 | Decision Tree | 2 | 0 | 0.79944072 | 0.78853047 | 0.78874001 | 279 | 13 | 10 |
| connectome | 4 | Random Forest | 2 | 0 | 0.7796003 | 0.80286738 | 0.77950113 | 279 | 13 | 10 |
| connectome | 5 | ANN - MLP | 2 | 0 | 0.74025663 | 0.74910394 | 0.74302243 | 279 | 13 | 10 |
| connectome | 6 | SVM Linear | 2 | 0 | 0.60410531 | 0.6344086 | 0.61183258 | 279 | 13 | 10 |
| connectome | 7 | Gaussian RBF | 2 | 0 | 0.5885103 | 0.61290323 | 0.5918526 | 279 | 13 | 10 |
| connectome | 8 | kNN | 2 | 0 | 0.53500919 | 0.56630824 | 0.53909893 | 279 | 13 | 10 |
| connectome | 9 | SVM RBF | 2 | 0 | 0.55261231 | 0.58064516 | 0.51914668 | 279 | 13 | 10 |
| connectome | 10 | Naive Bayes Gaussian | 2 | 0 | 0.60135407 | 0.53763441 | 0.51326904 | 279 | 13 | 10 |
| connectome | 11 | AdaBoost | 2 | 0 | 0.36974932 | 0.50179211 | 0.39322145 | 279 | 13 | 10 |
| heart_uci | 1 | SVM Linear | 5 | 0 | 0.8282814 | 0.82781457 | 0.82712512 | 302 | 19 | 2 |
| heart_uci | 2 | GSNAc | 5 | 0 | 0.8177077 | 0.81788079 | 0.81756799 | 302 | 19 | 2 |
| heart_uci | 3 | SVM RBF | 5 | 0 | 0.81807976 | 0.81788079 | 0.81724323 | 302 | 19 | 2 |
| heart_uci | 4 | kNN | 5 | 0 | 0.81441172 | 0.81456954 | 0.81445469 | 302 | 19 | 2 |
| heart_uci | 5 | Random Forest | 5 | 0 | 0.8047613 | 0.80463576 | 0.80468968 | 302 | 19 | 2 |
| heart_uci | 6 | xgboost | 5 | 0 | 0.80441942 | 0.80463576 | 0.80444767 | 302 | 19 | 2 |
| heart_uci | 7 | AdaBoost | 5 | 0 | 0.80471722 | 0.80463576 | 0.80395183 | 302 | 19 | 2 |
| heart_uci | 8 | Naive Bayes Gaussian | 5 | 0 | 0.79084902 | 0.78807947 | 0.78589099 | 302 | 19 | 2 |
| heart_uci | 9 | Gaussian RBF | 5 | 0 | 0.78114885 | 0.78145695 | 0.78099166 | 302 | 19 | 2 |
| heart_uci | 10 | ANN - MLP | 5 | 0 | 0.77123634 | 0.77152318 | 0.77130321 | 302 | 19 | 2 |
| heart_uci | 11 | Decision Tree | 5 | 0 | 0.76231928 | 0.7615894 | 0.76182067 | 302 | 19 | 2 |
| iris | 1 | GSNAc | 5 | 0 | 0.96019288 | 0.95973154 | 0.95970687 | 149 | 4 | 3 |
| iris | 2 | kNN | 5 | 0 | 0.95312706 | 0.95302013 | 0.95301054 | 149 | 4 | 3 |
| iris | 3 | Gaussian RBF | 5 | 0 | 0.95312706 | 0.95302013 | 0.95301054 | 149 | 4 | 3 |
| iris | 4 | Naive Bayes Gaussian | 5 | 0 | 0.95312706 | 0.95302013 | 0.95301054 | 149 | 4 | 3 |
| iris | 5 | SVM Linear | 5 | 0 | 0.95409934 | 0.95302013 | 0.95299136 | 149 | 4 | 3 |
| iris | 6 | SVM RBF | 5 | 0 | 0.94717992 | 0.94630872 | 0.94655905 | 149 | 4 | 3 |
| iris | 7 | ANN - MLP | 5 | 0 | 0.94684564 | 0.94630872 | 0.9465121 | 149 | 4 | 3 |
| iris | 8 | Random Forest | 5 | 0 | 0.93969601 | 0.93959732 | 0.93958498 | 149 | 4 | 3 |
| iris | 9 | Decision Tree | 5 | 0 | 0.93334649 | 0.93288591 | 0.93287221 | 149 | 4 | 3 |
| iris | 10 | xgboost | 5 | 0 | 0.92629503 | 0.9261745 | 0.9261745 | 149 | 4 | 3 |
| iris | 11 | AdaBoost | 5 | 0 | 0.92876754 | 0.9261745 | 0.92594769 | 149 | 4 | 3 |
| lymphoma | 1 | ANN - MLP | 2 | 0 | 0.92874504 | 0.92708333 | 0.92557367 | 96 | 4026 | 9 |
| lymphoma | 2 | SVM Linear | 2 | 0 | 0.91253472 | 0.92708333 | 0.90873506 | 96 | 4026 | 9 |
| lymphoma | 3 | GSNAc | 2 | 0 | 0.91354167 | 0.90625 | 0.90595934 | 96 | 4026 | 9 |
| lymphoma | 4 | Random Forest | 2 | 0 | 0.76379541 | 0.8125 | 0.76004143 | 96 | 4026 | 9 |
| lymphoma | 5 | kNN | 2 | 0 | 0.69380408 | 0.79166667 | 0.72693178 | 96 | 4026 | 9 |
| lymphoma | 6 | xgboost | 2 | 0 | 0.68853499 | 0.75 | 0.71435168 | 96 | 4026 | 9 |
| lymphoma | 7 | Decision Tree | 2 | 0 | 0.67173077 | 0.69791667 | 0.67683662 | 96 | 4026 | 9 |
| lymphoma | 8 | SVM RBF | 2 | 0 | 0.67225275 | 0.72916667 | 0.66313885 | 96 | 4026 | 9 |
| lymphoma | 9 | AdaBoost | 2 | 0 | 0.5921627 | 0.59375 | 0.56384857 | 96 | 4026 | 9 |
| lymphoma | 10 | Naive Bayes Gaussian | 2 | 0 | 0.47210249 | 0.57291667 | 0.45868578 | 96 | 4026 | 9 |
| lymphoma | 11 | Gaussian RBF | 2 | 0 | 0.00390625 | 0.0625 | 0.00735294 | 96 | 4026 | 9 |
| make_blobs | 1 | GSNAc | 5 | 0 | 0.79011605 | 0.79 | 0.789979 | 300 | 2 | 2 |
| make_blobs | 2 | SVM RBF | 5 | 0 | 0.78671764 | 0.78666667 | 0.78665718 | 300 | 2 | 2 |
| make_blobs | 3 | Gaussian RBF | 5 | 0 | 0.78671764 | 0.78666667 | 0.78665718 | 300 | 2 | 2 |
| make_blobs | 4 | ANN - MLP | 5 | 0 | 0.78344671 | 0.78333333 | 0.78331166 | 300 | 2 | 2 |
| make_blobs | 5 | Naive Bayes Gaussian | 5 | 0 | 0.77338193 | 0.77333333 | 0.77332326 | 300 | 2 | 2 |
| make_blobs | 6 | SVM Linear | 5 | 0 | 0.76671408 | 0.76666667 | 0.7666563 | 300 | 2 | 2 |
| make_blobs | 7 | Random Forest | 5 | 0 | 0.76041667 | 0.76 | 0.75990396 | 300 | 2 | 2 |
| make_blobs | 8 | kNN | 5 | 0 | 0.75337838 | 0.75333333 | 0.75332237 | 300 | 2 | 2 |
| make_blobs | 9 | AdaBoost | 5 | 0 | 0.75027809 | 0.75 | 0.74993054 | 300 | 2 | 2 |
| make_blobs | 10 | xgboost | 5 | 0 | 0.72682797 | 0.72666667 | 0.72661807 | 300 | 2 | 2 |
| make_blobs | 11 | Decision Tree | 5 | 0 | 0.71008403 | 0.71 | 0.709971 | 300 | 2 | 2 |
| make_moons | 1 | xgboost | 5 | 0 | 1 | 1 | 1 | 500 | 2 | 2 |
| make_moons | 1 | SVM RBF | 5 | 0 | 1 | 1 | 1 | 500 | 2 | 2 |
| make_moons | 1 | kNN | 5 | 0 | 1 | 1 | 1 | 500 | 2 | 2 |
| make_moons | 1 | ANN - MLP | 5 | 0 | 1 | 1 | 1 | 500 | 2 | 2 |
| make_moons | 1 | GSNAc | 5 | 0 | 1 | 1 | 1 | 500 | 2 | 2 |
| make_moons | 6 | Random Forest | 5 | 0 | 0.99800797 | 0.998 | 0.99799999 | 500 | 2 | 2 |
| make_moons | 7 | AdaBoost | 5 | 0 | 0.99603175 | 0.996 | 0.99599994 | 500 | 2 | 2 |
| make_moons | 8 | Decision Tree | 5 | 0 | 0.992 | 0.992 | 0.992 | 500 | 2 | 2 |
| make_moons | 9 | Gaussian RBF | 5 | 0 | 0.97400758 | 0.974 | 0.9739999 | 500 | 2 | 2 |
| make_moons | 10 | SVM Linear | 5 | 0 | 0.88 | 0.88 | 0.88 | 500 | 2 | 2 |
| make_moons | 11 | Naive Bayes Gaussian | 5 | 0 | 0.88 | 0.88 | 0.88 | 500 | 2 | 2 |
| pbc | 1 | GSNAc | 2 | 0 | 0.48355781 | 0.51449275 | 0.49473615 | 276 | 20 | 4 |
| pbc | 2 | SVM Linear | 2 | 0 | 0.48840515 | 0.49275362 | 0.48980787 | 276 | 20 | 4 |
| pbc | 3 | kNN | 2 | 0 | 0.4788951 | 0.47826087 | 0.47723596 | 276 | 20 | 4 |
| pbc | 4 | SVM RBF | 2 | 0 | 0.51028975 | 0.52173913 | 0.47067932 | 276 | 20 | 4 |
| pbc | 5 | Gaussian RBF | 2 | 0 | 0.44717419 | 0.46376812 | 0.4529763 | 276 | 20 | 4 |
| pbc | 6 | ANN - MLP | 2 | 0 | 0.44859107 | 0.46014493 | 0.4521886 | 276 | 20 | 4 |
| pbc | 7 | Random Forest | 2 | 0 | 0.43844792 | 0.44927536 | 0.43316496 | 276 | 20 | 4 |
| pbc | 8 | AdaBoost | 2 | 0 | 0.41598551 | 0.44927536 | 0.42899878 | 276 | 20 | 4 |
| pbc | 9 | xgboost | 2 | 0 | 0.41204652 | 0.4384058 | 0.42414068 | 276 | 20 | 4 |
| pbc | 10 | Decision Tree | 2 | 0 | 0.40410316 | 0.39130435 | 0.39491353 | 276 | 20 | 4 |
| pbc | 11 | Naive Bayes Gaussian | 2 | 0 | 0.49632381 | 0.24275362 | 0.25441271 | 276 | 20 | 4 |
| pokerhand | 1 | GSNAc | 2 | 0 | 0.43601171 | 0.452 | 0.44379952 | 250 | 75 | 6 |
| pokerhand | 2 | Gaussian RBF | 2 | 0 | 0.44160294 | 0.444 | 0.44152201 | 250 | 75 | 6 |
| pokerhand | 3 | SVM Linear | 2 | 0 | 0.4196 | 0.44 | 0.42881114 | 250 | 75 | 6 |
| pokerhand | 4 | Random Forest | 2 | 0 | 0.40452324 | 0.448 | 0.41876187 | 250 | 75 | 6 |
| pokerhand | 5 | SVM RBF | 2 | 0 | 0.41298902 | 0.464 | 0.4169253 | 250 | 75 | 6 |
| pokerhand | 6 | kNN | 2 | 0 | 0.39841652 | 0.448 | 0.41568168 | 250 | 75 | 6 |
| pokerhand | 7 | AdaBoost | 2 | 0 | 0.44670388 | 0.484 | 0.402 | 250 | 75 | 6 |
| pokerhand | 8 | ANN - MLP | 2 | 0 | 0.39706838 | 0.408 | 0.39418488 | 250 | 75 | 6 |
| pokerhand | 9 | Naive Bayes Gaussian | 2 | 0 | 0.37922608 | 0.404 | 0.38902412 | 250 | 75 | 6 |
| pokerhand | 10 | Decision Tree | 2 | 0 | 0.37221628 | 0.376 | 0.37285414 | 250 | 75 | 6 |
| pokerhand | 11 | xgboost | 2 | 0 | 0.34782517 | 0.376 | 0.36086908 | 250 | 75 | 6 |
| titanic | 1 | AdaBoost | 5 | 0 | 0.7704918 | 0.7704918 | 0.7704918 | 183 | 9 | 2 |
| titanic | 2 | GSNAc | 5 | 0 | 0.78258784 | 0.76502732 | 0.76995592 | 183 | 9 | 2 |
| titanic | 3 | kNN | 5 | 0 | 0.75832091 | 0.76502732 | 0.75964798 | 183 | 9 | 2 |
| titanic | 4 | Random Forest | 5 | 0 | 0.75438076 | 0.75956284 | 0.7560979 | 183 | 9 | 2 |
| titanic | 5 | xgboost | 5 | 0 | 0.74209682 | 0.7431694 | 0.74261018 | 183 | 9 | 2 |
| titanic | 6 | SVM Linear | 5 | 0 | 0.76607498 | 0.73224044 | 0.73968453 | 183 | 9 | 2 |
| titanic | 7 | Decision Tree | 5 | 0 | 0.74003435 | 0.73770492 | 0.7387799 | 183 | 9 | 2 |
| titanic | 8 | Gaussian RBF | 5 | 0 | 0.73586391 | 0.73224044 | 0.73385296 | 183 | 9 | 2 |
| titanic | 9 | SVM RBF | 5 | 0 | 0.74052351 | 0.72677596 | 0.73149593 | 183 | 9 | 2 |
| titanic | 10 | ANN - MLP | 5 | 0 | 0.70465024 | 0.71038251 | 0.70697112 | 183 | 9 | 2 |
| titanic | 11 | Naive Bayes Gaussian | 5 | 0 | 0.65922056 | 0.6284153 | 0.63798127 | 183 | 9 | 2 |
| wine | 1 | SVM RBF | 5 | 0 | 0.98330212 | 0.98314607 | 0.98318099 | 178 | 13 | 3 |
| wine | 2 | ANN - MLP | 5 | 0 | 0.98329512 | 0.98314607 | 0.98317318 | 178 | 13 | 3 |
| wine | 3 | Random Forest | 5 | 0 | 0.97210218 | 0.97191011 | 0.97185313 | 178 | 13 | 3 |
| wine | 4 | Naive Bayes Gaussian | 5 | 0 | 0.97210218 | 0.97191011 | 0.97185313 | 178 | 13 | 3 |
| wine | 5 | Gaussian RBF | 5 | 0 | 0.97326991 | 0.97191011 | 0.97174652 | 178 | 13 | 3 |
| wine | 6 | GSNAc | 5 | 0 | 0.96838914 | 0.96629213 | 0.96609165 | 178 | 13 | 3 |
| wine | 7 | SVM Linear | 5 | 0 | 0.96193882 | 0.96067416 | 0.96047577 | 178 | 13 | 3 |
| wine | 8 | kNN | 5 | 0 | 0.96193882 | 0.96067416 | 0.96047577 | 178 | 13 | 3 |
| wine | 9 | xgboost | 5 | 0 | 0.9496634 | 0.9494382 | 0.94941128 | 178 | 13 | 3 |
| wine | 10 | Decision Tree | 5 | 0 | 0.92169978 | 0.92134831 | 0.92137988 | 178 | 13 | 3 |
| wine | 11 | AdaBoost | 5 | 0 | 0.90705213 | 0.90449438 | 0.90464171 | 178 | 13 | 3 |
| weather_rain | 1 | GSNAc | 5 | 0 | 0.84598033 | 0.8539823 | 0.84717112 | 226 | 73 | 2 |
| weather_rain | 2 | Random Forest | 5 | 0 | 0.85124818 | 0.85840708 | 0.84631055 | 226 | 73 | 2 |
| weather_rain | 3 | xgboost | 5 | 0 | 0.82541352 | 0.83185841 | 0.82790119 | 226 | 73 | 2 |
| weather_rain | 4 | AdaBoost | 5 | 0 | 0.8057915 | 0.80973451 | 0.8075962 | 226 | 73 | 2 |
| weather_rain | 5 | SVM RBF | 5 | 0 | 0.84355736 | 0.83628319 | 0.8029813 | 226 | 73 | 2 |
| weather_rain | 6 | ANN - MLP | 5 | 0 | 0.78695658 | 0.80088496 | 0.79159699 | 226 | 73 | 2 |
| weather_rain | 7 | SVM Linear | 5 | 0 | 0.76960674 | 0.73451327 | 0.74760843 | 226 | 73 | 2 |
| weather_rain | 8 | kNN | 5 | 0 | 0.76018119 | 0.7920354 | 0.74544901 | 226 | 73 | 2 |
| weather_rain | 9 | Gaussian RBF | 5 | 0 | 0.73462033 | 0.75221239 | 0.74186889 | 226 | 73 | 2 |
| weather_rain | 10 | Decision Tree | 5 | 0 | 0.73831405 | 0.73451327 | 0.73635755 | 226 | 73 | 2 |
| weather_rain | 11 | Naive Bayes Gaussian | 5 | 0 | 0.72830343 | 0.54424779 | 0.5807801 | 226 | 73 | 2 |
| voice | 1 | SVM RBF | 5 | 0 | 0.97076586 | 0.97046414 | 0.9704594 | 474 | 20 | 2 |
| voice | 2 | ANN - MLP | 5 | 0 | 0.96627793 | 0.96624473 | 0.96624412 | 474 | 20 | 2 |
| voice | 3 | xgboost | 5 | 0 | 0.96414328 | 0.96413502 | 0.96413486 | 474 | 20 | 2 |
| voice | 4 | GSNAc | 5 | 0 | 0.96232163 | 0.96202532 | 0.96201923 | 474 | 20 | 2 |
| voice | 5 | Random Forest | 5 | 0 | 0.95998932 | 0.95991561 | 0.95991401 | 474 | 20 | 2 |
| voice | 6 | Decision Tree | 5 | 0 | 0.95783851 | 0.95780591 | 0.95780516 | 474 | 20 | 2 |
| voice | 7 | AdaBoost | 5 | 0 | 0.95570432 | 0.9556962 | 0.95569601 | 474 | 20 | 2 |
| voice | 8 | SVM Linear | 5 | 0 | 0.95589912 | 0.9556962 | 0.95569127 | 474 | 20 | 2 |
| voice | 9 | Gaussian RBF | 5 | 0 | 0.9563543 | 0.9556962 | 0.95568022 | 474 | 20 | 2 |
| voice | 10 | kNN | 5 | 0 | 0.944375 | 0.94303797 | 0.9429951 | 474 | 20 | 2 |
| voice | 11 | Naive Bayes Gaussian | 5 | 0 | 0.89521839 | 0.89451477 | 0.8944678 | 474 | 20 | 2 |
| heart_kaggle | 1 | Random Forest | 5 | 0 | 0.82642532 | 0.82943144 | 0.82708667 | 299 | 12 | 2 |
| heart_kaggle | 2 | SVM Linear | 5 | 0 | 0.82591981 | 0.82943144 | 0.82588366 | 299 | 12 | 2 |
| heart_kaggle | 3 | GSNAc | 5 | 0 | 0.82327197 | 0.82608696 | 0.81930349 | 299 | 12 | 2 |
| heart_kaggle | 4 | xgboost | 5 | 0 | 0.81846147 | 0.81939799 | 0.81889011 | 299 | 12 | 2 |
| heart_kaggle | 5 | SVM RBF | 5 | 0 | 0.80453275 | 0.80936455 | 0.80393585 | 299 | 12 | 2 |
| heart_kaggle | 6 | AdaBoost | 5 | 0 | 0.79349434 | 0.79598662 | 0.79450013 | 299 | 12 | 2 |
| heart_kaggle | 7 | ANN - MLP | 5 | 0 | 0.78717395 | 0.78595318 | 0.78652669 | 299 | 12 | 2 |
| heart_kaggle | 8 | Naive Bayes Gaussian | 5 | 0 | 0.76154777 | 0.76923077 | 0.75395658 | 299 | 12 | 2 |
| heart_kaggle | 9 | Decision Tree | 5 | 0 | 0.75131043 | 0.74916388 | 0.75015976 | 299 | 12 | 2 |
| heart_kaggle | 10 | kNN | 5 | 0 | 0.72736382 | 0.73913043 | 0.71310615 | 299 | 12 | 2 |
| heart_kaggle | 11 | Gaussian RBF | 5 | 0 | 0.70059442 | 0.71906355 | 0.69103739 | 299 | 12 | 2 |
| breast_cancer_wisconsin | 1 | SVM RBF | 5 | 0 | 0.97714393 | 0.9771529 | 0.97711929 | 569 | 30 | 2 |
| breast_cancer_wisconsin | 2 | SVM Linear | 5 | 0 | 0.9754017 | 0.97539543 | 0.9753468 | 569 | 30 | 2 |
| breast_cancer_wisconsin | 3 | ANN - MLP | 5 | 0 | 0.9754017 | 0.97539543 | 0.9753468 | 569 | 30 | 2 |
| breast_cancer_wisconsin | 4 | xgboost | 5 | 0 | 0.97022854 | 0.97012302 | 0.97001729 | 569 | 30 | 2 |
| breast_cancer_wisconsin | 5 | GSNAc | 5 | 0 | 0.96840701 | 0.96836555 | 0.96827032 | 569 | 30 | 2 |
| breast_cancer_wisconsin | 6 | Gaussian RBF | 5 | 0 | 0.96682353 | 0.96660808 | 0.96645384 | 569 | 30 | 2 |
| breast_cancer_wisconsin | 7 | AdaBoost | 5 | 0 | 0.96682353 | 0.96660808 | 0.96645384 | 569 | 30 | 2 |
| breast_cancer_wisconsin | 8 | kNN | 5 | 0 | 0.96560525 | 0.96485062 | 0.96458848 | 569 | 30 | 2 |
| breast_cancer_wisconsin | 9 | Random Forest | 5 | 0 | 0.96143103 | 0.96133568 | 0.96117811 | 569 | 30 | 2 |
| breast_cancer_wisconsin | 10 | Naive Bayes Gaussian | 5 | 0 | 0.92952129 | 0.92970123 | 0.92956229 | 569 | 30 | 2 |
| breast_cancer_wisconsin | 11 | Decision Tree | 5 | 0 | 0.91955185 | 0.91915641 | 0.91930646 | 569 | 30 | 2 |
| pima_diabetes | 1 | SVM Linear | 5 | 0 | 0.7696127 | 0.77473958 | 0.76773259 | 768 | 8 | 2 |
| pima_diabetes | 2 | Random Forest | 5 | 0 | 0.75718009 | 0.76302083 | 0.75710814 | 768 | 8 | 2 |
| pima_diabetes | 3 | SVM RBF | 5 | 0 | 0.75843191 | 0.76432292 | 0.75596485 | 768 | 8 | 2 |
| pima_diabetes | 4 | ANN - MLP | 5 | 0 | 0.75173806 | 0.75651042 | 0.75297781 | 768 | 8 | 2 |
| pima_diabetes | 5 | Naive Bayes Gaussian | 5 | 0 | 0.7501179 | 0.75390625 | 0.75141964 | 768 | 8 | 2 |
| pima_diabetes | 6 | Gaussian RBF | 5 | 0 | 0.74640198 | 0.75260417 | 0.74707324 | 768 | 8 | 2 |
| pima_diabetes | 7 | GSNAc | 5 | 0 | 0.7425518 | 0.74869792 | 0.74355442 | 768 | 8 | 2 |
| pima_diabetes | 8 | AdaBoost | 5 | 0 | 0.73900463 | 0.74609375 | 0.73925036 | 768 | 8 | 2 |
| pima_diabetes | 9 | kNN | 5 | 0 | 0.73184212 | 0.73958333 | 0.73203016 | 768 | 8 | 2 |
| pima_diabetes | 10 | xgboost | 5 | 0 | 0.72580811 | 0.73046875 | 0.72745655 | 768 | 8 | 2 |
| pima_diabetes | 11 | Decision Tree | 5 | 0 | 0.68870163 | 0.69270833 | 0.69040039 | 768 | 8 | 2 |
| caravan | 1 | SVM RBF | 5 | 0 | 0.88356175 | 0.93997965 | 0.91089796 | 983 | 141 | 2 |
| caravan | 2 | xgboost | 5 | 0 | 0.89656497 | 0.92472024 | 0.90891465 | 983 | 141 | 2 |
| caravan | 3 | GSNAc | 5 | 0 | 0.88333124 | 0.93591048 | 0.90886104 | 983 | 141 | 2 |
| caravan | 3 | kNN | 5 | 0 | 0.88333124 | 0.93591048 | 0.90886104 | 983 | 141 | 2 |
| caravan | 5 | AdaBoost | 5 | 0 | 0.89297625 | 0.92268566 | 0.90645289 | 983 | 141 | 2 |
| caravan | 6 | Random Forest | 5 | 0 | 0.88304043 | 0.93082401 | 0.90630283 | 983 | 141 | 2 |
| caravan | 7 | Gaussian RBF | 5 | 0 | 0.89056413 | 0.90437436 | 0.89728472 | 983 | 141 | 2 |
| caravan | 8 | ANN - MLP | 5 | 0 | 0.88673667 | 0.90742625 | 0.89678895 | 983 | 141 | 2 |
| caravan | 9 | SVM Linear | 5 | 0 | 0.88602973 | 0.90233978 | 0.89402139 | 983 | 141 | 2 |
| caravan | 10 | Decision Tree | 5 | 0 | 0.88421782 | 0.86775178 | 0.8758531 | 983 | 141 | 2 |
| caravan | 11 | Naive Bayes Gaussian | 5 | 0 | 0.90203161 | 0.491353 | 0.60977683 | 983 | 141 | 2 |
| digits | 1 | SVM RBF | 5 | 0 | 0.98070779 | 0.98052309 | 0.98053352 | 1797 | 64 | 10 |
| digits | 2 | SVM Linear | 5 | 0 | 0.97899151 | 0.97885364 | 0.97878567 | 1797 | 64 | 10 |
| digits | 3 | kNN | 5 | 0 | 0.97691292 | 0.97662771 | 0.97661867 | 1797 | 64 | 10 |
| digits | 4 | Random Forest | 5 | 0 | 0.97612573 | 0.97607123 | 0.97599487 | 1797 | 64 | 10 |
| digits | 5 | GSNAc | 5 | 0 | 0.97601588 | 0.97551475 | 0.97549691 | 1797 | 64 | 10 |
| digits | 6 | ANN - MLP | 5 | 0 | 0.97108837 | 0.97106288 | 0.97097428 | 1797 | 64 | 10 |
| digits | 7 | Gaussian RBF | 5 | 0 | 0.9683771 | 0.9671675 | 0.96738376 | 1797 | 64 | 10 |
| digits | 8 | xgboost | 5 | 0 | 0.96482034 | 0.96438509 | 0.96441568 | 1797 | 64 | 10 |
| digits | 9 | Decision Tree | 5 | 0 | 0.85301951 | 0.85197551 | 0.85226648 | 1797 | 64 | 10 |
| digits | 10 | Naive Bayes Gaussian | 5 | 0 | 0.82612428 | 0.78575403 | 0.78403417 | 1797 | 64 | 10 |
| digits | 11 | AdaBoost | 5 | 0 | 0.29821039 | 0.28436283 | 0.26332106 | 1797 | 64 | 10 |
| forest_type | 1 | GSNAc | 5 | 0 | 0.62915374 | 0.6277666 | 0.6192002 | 497 | 52 | 7 |
| forest_type | 2 | Decision Tree | 5 | 0 | 0.58463495 | 0.57142857 | 0.57126597 | 497 | 52 | 7 |
| forest_type | 3 | SVM Linear | 5 | 0 | 0.57304404 | 0.5694165 | 0.56918821 | 497 | 52 | 7 |
| forest_type | 4 | SVM RBF | 5 | 0 | 0.57535574 | 0.5694165 | 0.56765729 | 497 | 52 | 7 |
| forest_type | 5 | Random Forest | 5 | 0 | 0.57730744 | 0.5694165 | 0.56747796 | 497 | 52 | 7 |
| forest_type | 6 | Gaussian RBF | 5 | 0 | 0.57339742 | 0.57142857 | 0.56565417 | 497 | 52 | 7 |
| forest_type | 7 | ANN - MLP | 5 | 0 | 0.56638043 | 0.56338028 | 0.55955553 | 497 | 52 | 7 |
| forest_type | 8 | xgboost | 5 | 0 | 0.56191111 | 0.55331992 | 0.55447207 | 497 | 52 | 7 |
| forest_type | 9 | kNN | 5 | 0 | 0.55776888 | 0.54325956 | 0.54582772 | 497 | 52 | 7 |
| forest_type | 10 | AdaBoost | 5 | 0 | 0.4589272 | 0.4305835 | 0.43193415 | 497 | 52 | 7 |
| forest_type | 11 | Naive Bayes Gaussian | 5 | 0 | 0.49577646 | 0.46881288 | 0.41159488 | 497 | 52 | 7 |
| covid | 1 | GSNAc | 2 | 0 | 0.78208189 | 0.77981651 | 0.77003411 | 436 | 37 | 2 |
| covid | 2 | xgboost | 2 | 0 | 0.76546321 | 0.76834862 | 0.7659523 | 436 | 37 | 2 |
| covid | 3 | SVM Linear | 2 | 0 | 0.76812836 | 0.7706422 | 0.76423887 | 436 | 37 | 2 |
| covid | 4 | Random Forest | 2 | 0 | 0.7579255 | 0.76146789 | 0.7557965 | 436 | 37 | 2 |
| covid | 5 | ANN - MLP | 2 | 0 | 0.7507113 | 0.75229358 | 0.75135606 | 436 | 37 | 2 |
| covid | 6 | SVM RBF | 2 | 0 | 0.7613499 | 0.76146789 | 0.75087029 | 436 | 37 | 2 |
| covid | 7 | Gaussian RBF | 2 | 0 | 0.76124417 | 0.75688073 | 0.74198306 | 436 | 37 | 2 |
| covid | 8 | AdaBoost | 2 | 0 | 0.73897077 | 0.74311927 | 0.73940362 | 436 | 37 | 2 |
| covid | 9 | Naive Bayes Gaussian | 2 | 0 | 0.72924339 | 0.73394495 | 0.72963201 | 436 | 37 | 2 |
| covid | 10 | Decision Tree | 2 | 0 | 0.70648813 | 0.7087156 | 0.70741627 | 436 | 37 | 2 |
| covid | 11 | kNN | 2 | 0 | 0.67047783 | 0.67889908 | 0.65060443 | 436 | 37 | 2 |

# Appendix II

## Graphical User Interface (GUI) of GSNAc Classifier

We have developed a practical GUI in Microsoft Excel for experimenting with GSNAc. This GUI (as illustrated below) allows batch processing of scenarios. Users can specify the dataset and desired parameters for the experiments, and via GUI GSNAc processes scenarios and produces detailed outputs.

## A-II- Figure 1 Output Report of GSNAc

GSNAc produces very detailed outputs of its experiments. After every run of GSNAc, detailed file outputs for every fold and for every sample are produced including adjacency matrices, raw graph files, GCMs and overall prediction result graphs. To simplify the utilization of this excess information, it also produces a simple HTML webpage as a summary report. This report (as illustrated below) compiles key information during the classification such as average feature importance for data, comparison of performance with other classifiers, parameters used, overall classification performance on the same graph for each classifier (in order to visually compare the performance on sample-level).

| 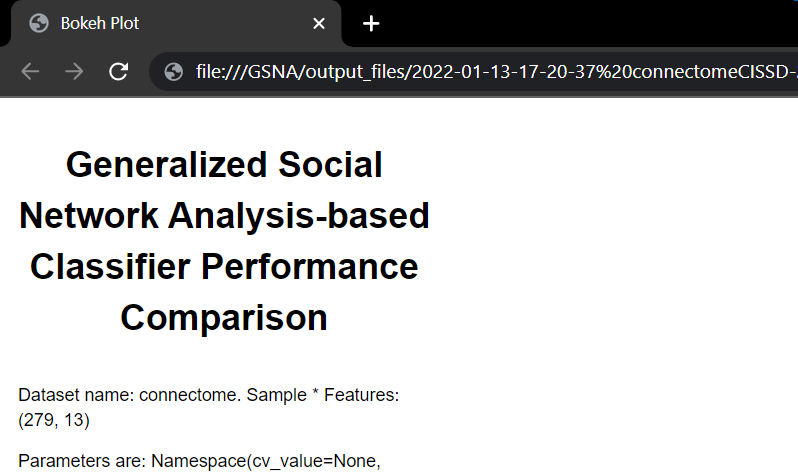  A-II- Figure 2 Classifier Performance Comparison first screen  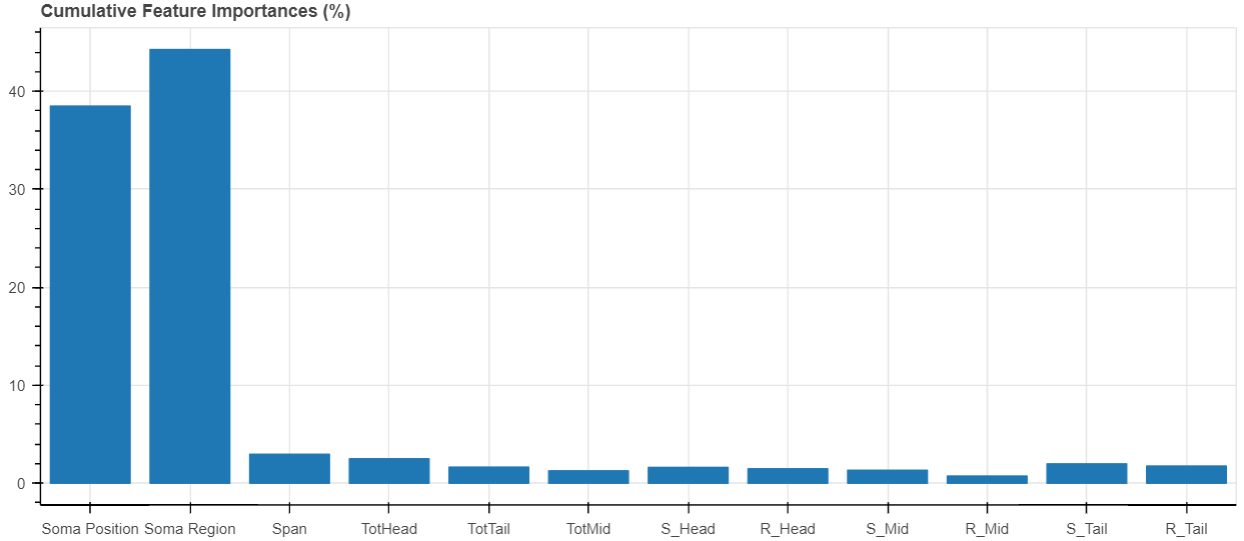  A-II- Figure 3 Cumulative Feature Importance  A-II- Table 1 Classifier Performance Comparison  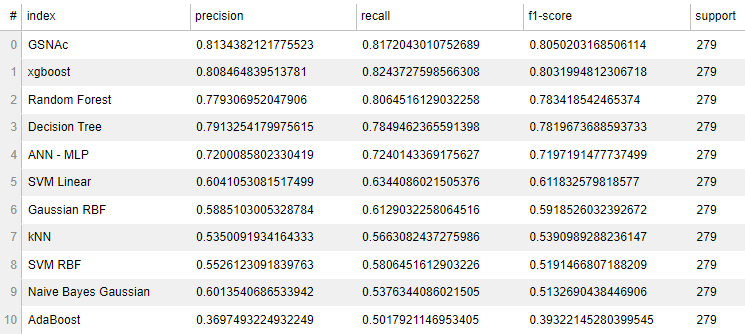  A-II- Table 2 Classification Report by Each Sample  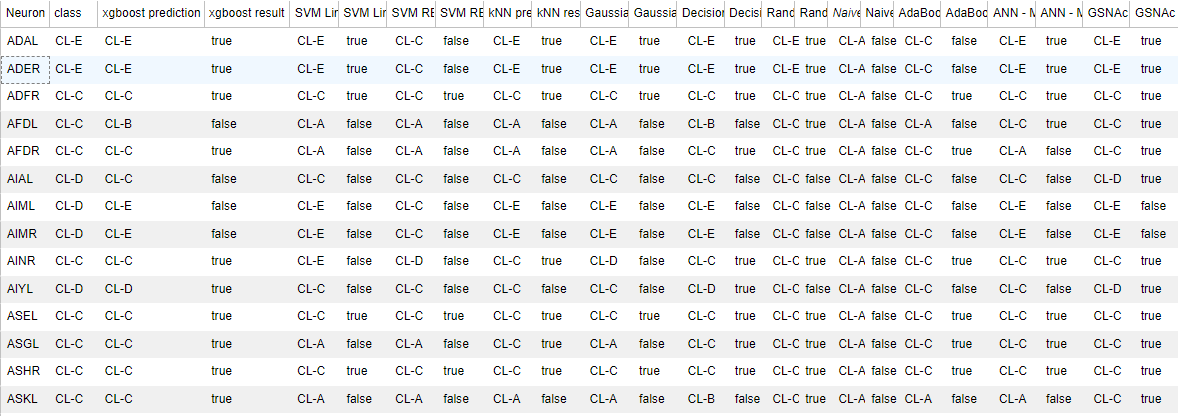  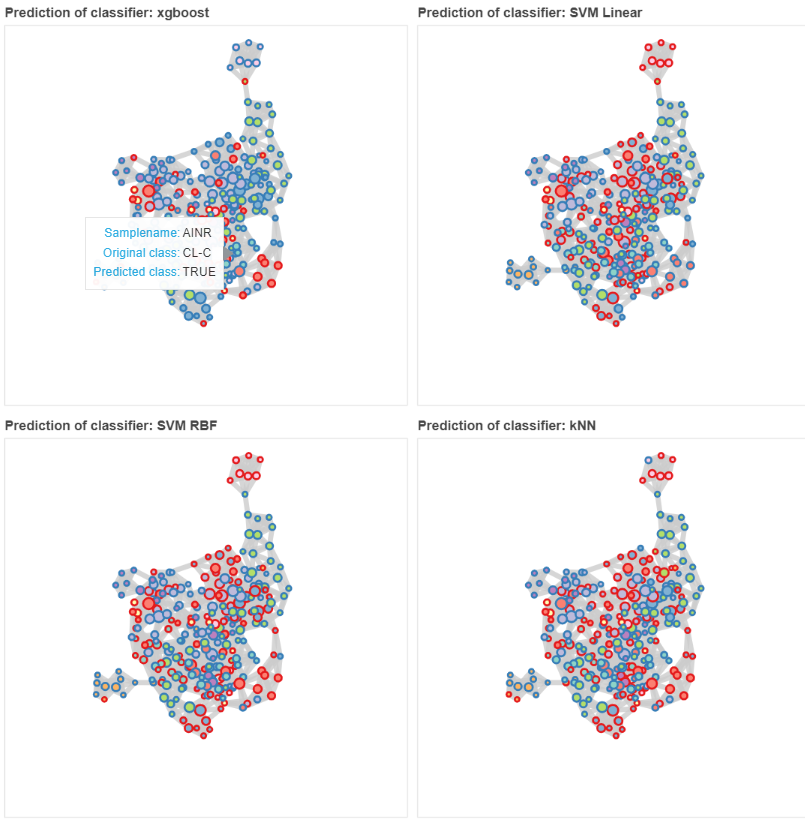  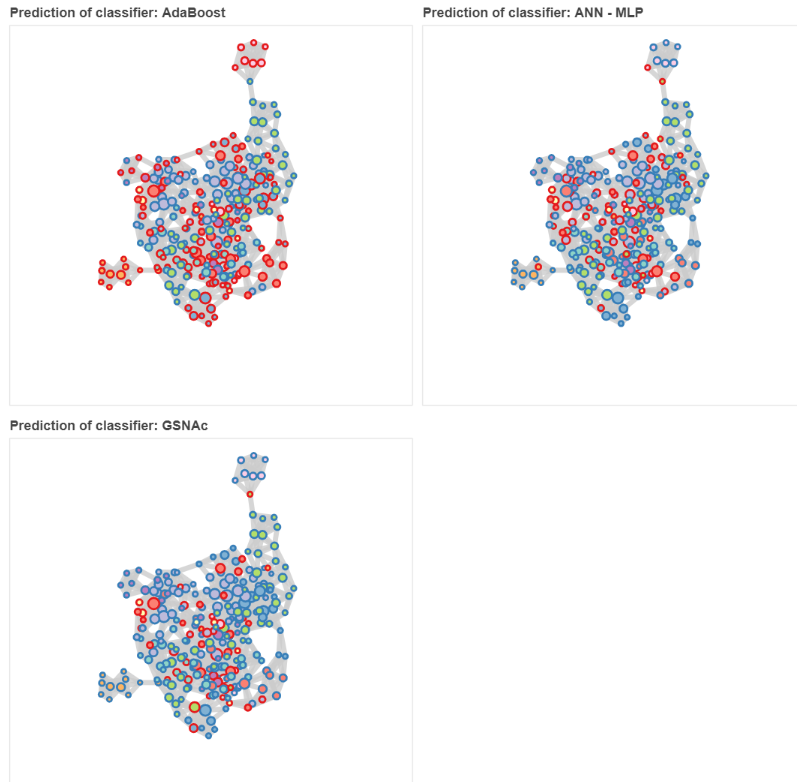  A-II- Figure 4 Overall Classification Graphs by Each Classifier |
| --- |
